# Advantages, Utility, and Efficiency of Geophysical Well Log Cross-Plots to Evaluate the Petrophysical Properties of Oil Reservoirs

**DOI:** 10.1021/acsomega.6c01355

**Published:** 2026-09-27

**Authors:** Abel Carrasquilla, Herson da Rocha, Rodrigo da Silva

**Affiliations:** † Petroleum Engineering and Exploration Laboratory, 28109 Universidade Estadual do Norte Fluminense Darcy Ribeiro, Av. Alberto Lamego, 2000, Parque Califórnia, Campos dos Goytacazes, 28013-602 Rio de Janeiro, Brazil; ‡ Polytechnic Institute, Campus Macaé, Universidade Federal do Rio de Janeiro, Cidade Universitária, Ilha do Fundão, Av. Pedro Calmon, 550, Zip Code 21941-901 Rio de Janeiro, Brazil

## Abstract

Core sample measurements and well logs were integrated to evaluate reservoir petrophysical properties and develop more precise models for oil production. These logs are frequently used to generate cross-plots that reveal underlying patterns and assist in reservoir classification. In this study, cross-plots helped characterize the petrophysics of Namorado sandstone and Enchova limestone reservoirs in the Campos Basin, southeastern Brazil. Hingle, Pickett, Buckles, Neutron-Density, and M-N cross-plots were created from basic logs obtained from a single borehole in each oilfield. The estimated sonic porosity aligns with laboratory measurements of effective porosity and is more suitable for reservoir calculations, offering nearly continuous, full-interval estimates. Results show that transit time in the Enchova reservoir is slightly higher than in Namorado, while formation water resistivity, matrix density, and irreducible water saturation are similar. The limestone reservoir holds more water, and a higher formation water resistivity suggests it contains more oil in its pores. This study underscores the usefulness and efficiency of cross-plots for petrophysical assessment, highlighting their role as vital tools for quickly visualizing data and understanding relationships among multiple properties.

## Graphical abstract

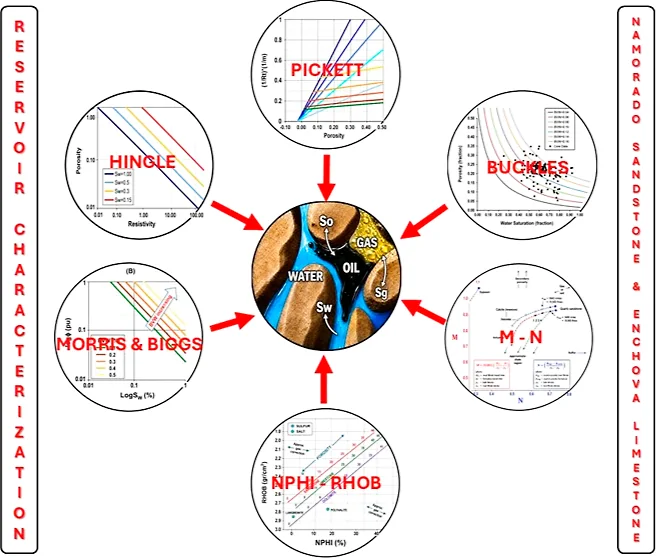

## Introduction

Petrophysical cross-plots gained prominence in the oil and gas industry between the late 1950s and the 1960s, becoming essential tools for integrating multiple well logs and for improving estimates of porosity, lithology, fluid type, and reservoir quality compared with single-log interpretations. Despite their long history, cross-plots remain widely used because they provide an effective visual framework for identifying relationships, trends, and clusters within petrophysical data sets.^1^ The novelty of the present study does not lie in the cross-plot concept itself, but in its integrated, multiscale, and systematic application to combine conventional well logs, derived petrophysical parameters, and geological information for enhanced reservoir characterization. By jointly analyzing variations in gamma ray, resistivity, density, neutron porosity, sonic logs, and fluid-saturation indicators, the proposed approach transforms conventional scatter plots into an integrated interpretation workflow capable of highlighting subtle lithological variations, fluid effects, and reservoir-quality patterns that may not be evident when individual logs or isolated cross-plots are examined. Furthermore, the visual clustering of wireline-log data, supported by geological and core information, improves the discrimination of reservoir facies and provides a more consistent assessment of subsurface heterogeneity and hydrocarbon potential.^2^

Cross-plots using logs help distinguish minerals and lithofacies in the subsurface. They form distinct clusters for different rock types and fluids, aiding visual identification and estimation of reservoir parameters.^3^ They are also vital for mapping hydrocarbon reservoirs and locating fluid-bearing zones, which are essential for evaluating reservoir quality and economic extraction potential.^4^ Data integration, combining information from multiple well logs in cross-plots, provides a clearer subsurface view than single logs alone. They are vital for understanding the relationship between reservoir properties and oil production, as well as for guiding reservoir management decisions.^5^ Practical applications include analyzing porosity, fluid saturation, and combining logs to reveal subsurface formations.^6^

Recent studies from hydrocarbon provinces worldwide demonstrate that petrophysical cross-plots, introduced decades ago, remain relevant and adaptable tools for modern reservoir characterization. For instance, Cao et al.^2^ introduced an enhanced visualization method using augmented-dimensional cross-plots to integrate heterogeneous geological, well-log, and well-testing data from multiple wells in a sandstone oil and gas reservoir in the Sichuan Basin, China, one of the country’s major hydrocarbon-producing regions. Similarly, Austin et al.^5^ and Bello et al.^1^ combined petrophysical well-log cross-plots with rock-physics models to distinguish lithologies, identify gas-bearing sands, and discriminate fluid types within the Coastal Swamp depobelt of the Niger Delta Basin, Nigeria. Haijing et al.^7^ further demonstrated the continued evolution of this classical approach by integrating conventional and advanced measurements, including nuclear spectroscopy, nuclear magnetic resonance, and dielectric logs, across multiple cross-plots to characterize complex mineral assemblages and identify organic matter. Collectively, these recent applications across Asia and Africa show that, despite their historical origins, cross-plots continue to evolve and remain effective for integrating increasingly diverse data sets and addressing complex petrophysical problems in different geological settings worldwide.

More recently, cross-plot methods have been integrated into artificial intelligence (AI) techniques and automated interpretation workflows for advanced reservoir analyses. Thus, researchers increasingly incorporate advances in machine learning (ML) methods with cross-plots to enhance interpretation, automate feature detection, and manage multidimensional, multiwell data sets. ML turns cross-plots from basic visualization tools into predictive, high-dimensional analytics platforms. For example, using cross-plots for a comprehensive evaluation of well-logging, geology, mud-logging, and well-testing data, or analyzing data from multiple wells and layers more thoroughly, including expanding the traditional cross-plot into multiple dimensions. Although AI and digital processing are widespread, manual cross-plotting remains useful for quick insights.^2^

As a highly discussed topic nowadays, CO_2_ capture and utilization are considered essential strategies to improve and support the storage of this gas, thereby reducing its emissions. Geological storage is a promising method that can help decrease greenhouse gas production. However, effective storage in geological underground formations requires understanding the primary storage techniques and trapping mechanisms.^8^ Thus, cross-plots of logs can be very effective tools for quickly evaluating potential geological storage reservoirs for this gas. They allow a ″quick-look″ assessment of key parameters such as lithology, porosity, fluid type, and caprock integrity without the need for time-consuming core analysis or complex numerical modeling during the initial screening phase.^6^ For this reason, many studies in this research line are available in the literature.^9–14^

This study examined data from Brazil’s Campos Basin to assess how well cross-plots characterize reservoirs in the Namorado and Enchova oilfields, with a focus on their siliciclastic and carbonate formations. Unlike previous research, this work mainly aimed to compare these two reservoir types across different offshore fields and highlight their differences. It advances formation evaluation by offering a comprehensive, educational, and current workflow that extends beyond simple geophysical log analysis. Its main contributions include integrating established interpretive methods, comparing results, visualizing patterns, and improving assessment accuracy in complex reservoirs. The key innovation transforms traditional petrophysical cross-plots into a holistic multivariate interpretation approach, enabling the detection of subtle reservoir patterns and improved discrimination of lithology and fluids. The results demonstrate that cross-plots are highly effective for quickly visualizing and interpreting complex relationships among reservoir properties such as porosity, water saturation, lithology, and hydrocarbon potential.

## Geological Context

The Campos Basin, located along the Brazilian margin (Figure 1), originated during the breakup of the Gondwana supercontinent, as the tectonic plates underwent Mesozoic adjustments, leading to the division of several rigid continental blocks.^15^

**Figure 1:**
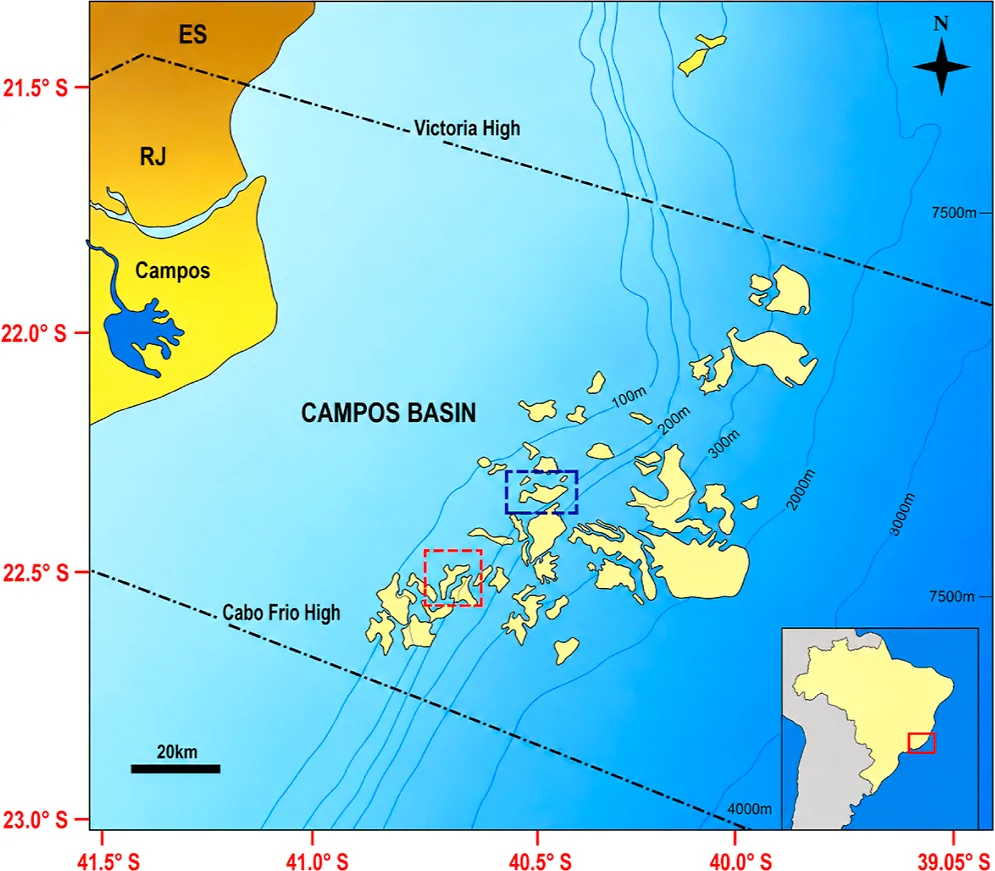
Location map of the Campos Basin, southeastern Brazil, with boundaries and regional geological features based on Bruhn et al.^18^ and oilfield locations compiled from publicly available ANP^19^ data. Map created by the authors.

During the Albian-Cenomanian period, the basin was characterized by marine conditions, leading to the deposition of clastic and oolitic carbonates in the Macaé Formation, particularly in the Quissamã Member (Figure 2), which exhibits significant dolomitization and serves as a reservoir for Albian carbonates.^16^ The Outeiro Member presents a vertical sequence of calcilutite, marl, and shale. In more proximal areas, the Namorado Member features turbiditic sandstone, while the Goitacás Member comprises poorly sorted conglomerate and sandstone, serving as turbidite reservoirs over the Albian formations and correlating with the Middle/Upper Cenozoic of the Upper Macaé Group.^17^

**Figure 2:**
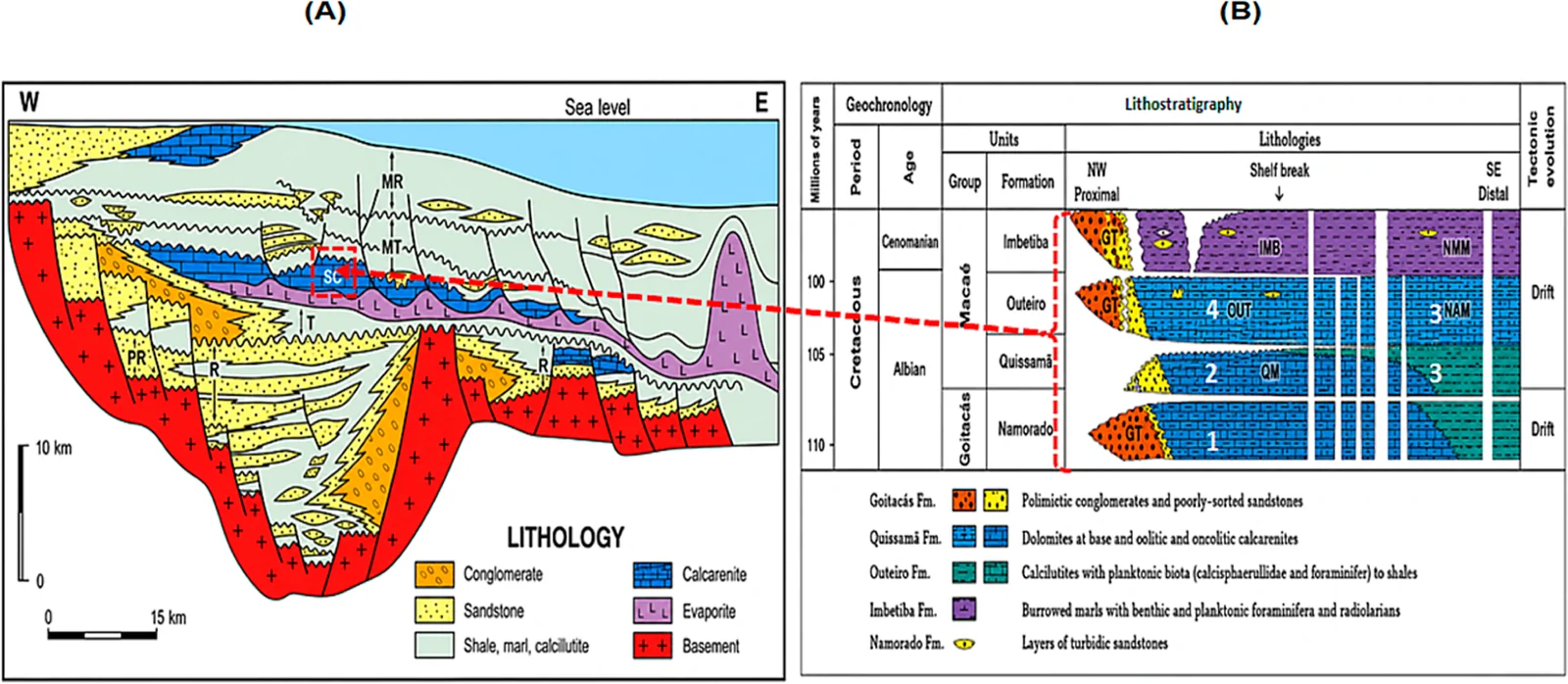
Simplified geological evolution and lithostratigraphic framework of the Campos Basin, independently compiled and redrawn after Bruhn et al.,^18^ Winter et al.,^20^ Rockett et al.,^21^ and Okubo et al.^22^ (A) The four main megasequences are PR (prerift), R (rift), T (transitional, including the evaporite section), SC (shallow carbonate), MT (marine transgressive), and MR (marine regressive). (B) A closer look at the stratigraphy of the Campos Basin’s postsalt carbonates, emphasizing the studied region, the dolomites, and the calcarenites of the Macaé Group (Goitacás Formation), which is formed by Quissamã (1), Outeiro (2), Imbetiba (3), and Namorado (4) Formations.

## Materials and Methods

The research focused on analyzing basic logs from the Enchova and Namorado oilfields, specifically examining GR, NPHI, DT, RHOB, and RT data.^19^ To validate log-derived porosity values, laboratory measurements were also used.^23^ The Interactive Petrophysics (IP) software facilitated data interpretation and the creation of graphical log representations.^24^ Several mathematical calculations were performed using MATLAB.^25^ Initial analysis identified reservoir zones, followed by using various cross-plots, including those for evaluating petrophysical properties.^26–30^ Figure 3 illustrates the study’s workflow.

**Figure 3:**
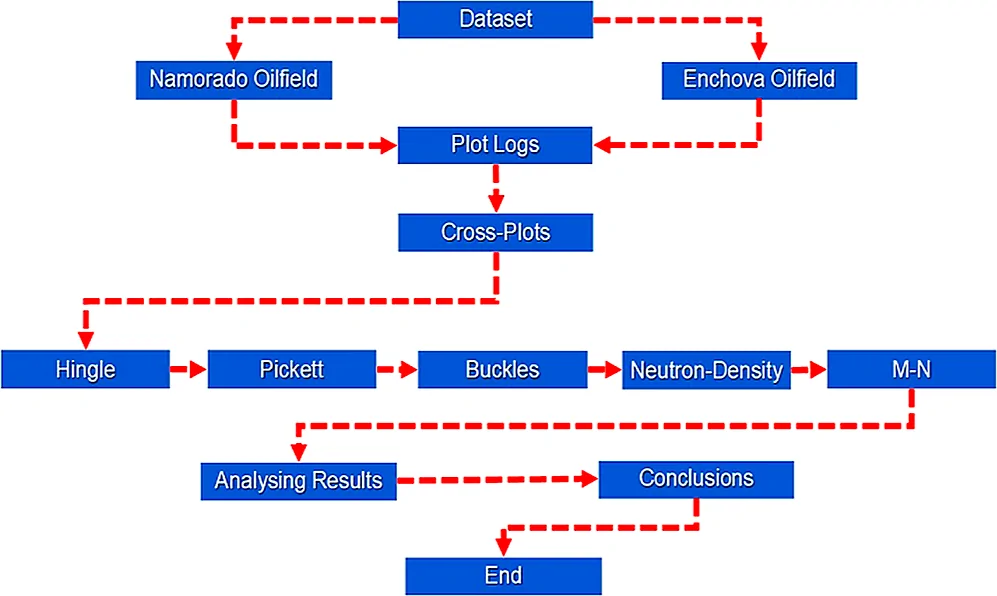
Workflow followed in this work.

A Hingle cross-plot^26^ is a petrophysical analysis tool that visually connects porosity (ϕ) and resistivity (RT) log data using Archie’s equation.^31^ This plot uses a nonlinear graph to depict lines of constant water saturation (*S*_*W*_), aiding in determining rock-matrix properties such as water formation resistivity (*R*_*W*_), rock-matrix density (ρ_MA_), and travel time (DT_MA_). The cross-plot can also be represented using density (RHOB) and delay time (DT) logs on the *x*-axis, and RT on the *y*-axis, with logarithmic scales. In this setup, DT_MA_ is marked by the intersection of the waterline S_W_ = 1.0 dec (decimal fraction representing a scale from 0.0 to 1.0) and RT = ∞ ohm·m (Ω·m) with the DT axis.

The ρ_MA_ point, where the waterline intersects the RHOB axis, provides further insights, along with R_W_, the waterline’s y-intercept, and Archie’s cementation coefficient (m), which highlight different levels of S_W_ (Figure 4).

**Figure 4:**
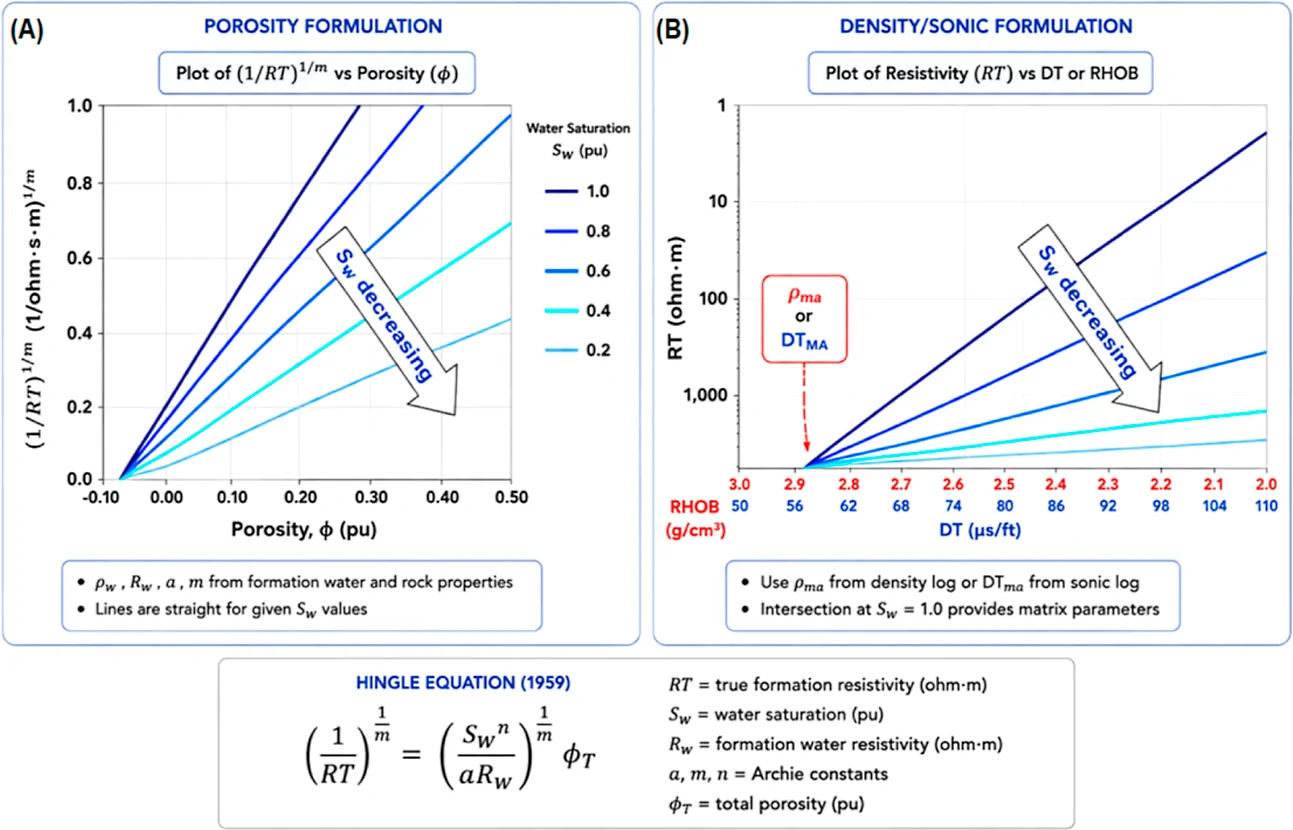
Cross-plots for estimating SW and matrix parameters (ρMA and DTMA). Adapted from the method proposed by Hingle^26^ and independently compiled and redrawn after Krygowski and Cluff:^30^ (A) Original: (pu) vs (1/RT)­1/m (mhos.m). (B) Modified: RHOB (gr/cm^3^) vs RT (Ω·m) and DT (μs/feet) vs RT (Ω·m).

A Pickett cross-plot^27^ uses the RT versus ϕ plot from well logs to compare Sw across different reservoir sections, effectively visualizing the Archie equation^31^ as a straight line (Figure 5).

**Figure 5:**
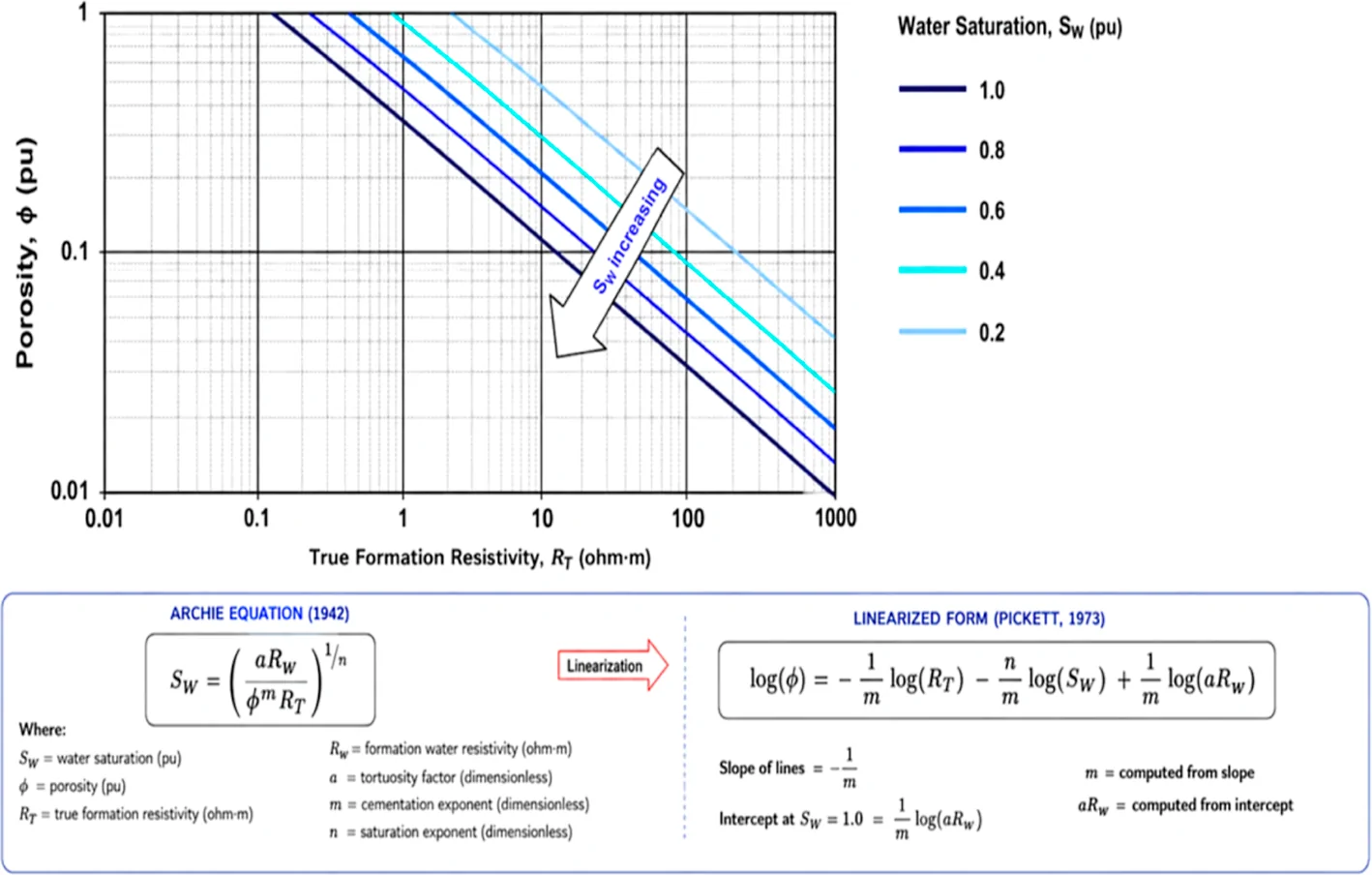
Pickett cross-plot for graphical interpretation of S_W_ and Archie parameters. Adapted from the method proposed by Archie^31^ and Pickett,^29^ and independently compiled and redrawn after Krygowski and Cluff.^30^

Buckles^28^ initially proposed a hyperbolic cross-plot of Sw versus ϕ, assuming a constant bulk-volume-water (BVW = ϕ × Sw) curve, while Morris and Biggs^29^ linearized this equation to enhance reservoir measurement analysis and enable BVW estimation (Figure 6).

**Figure 6:**
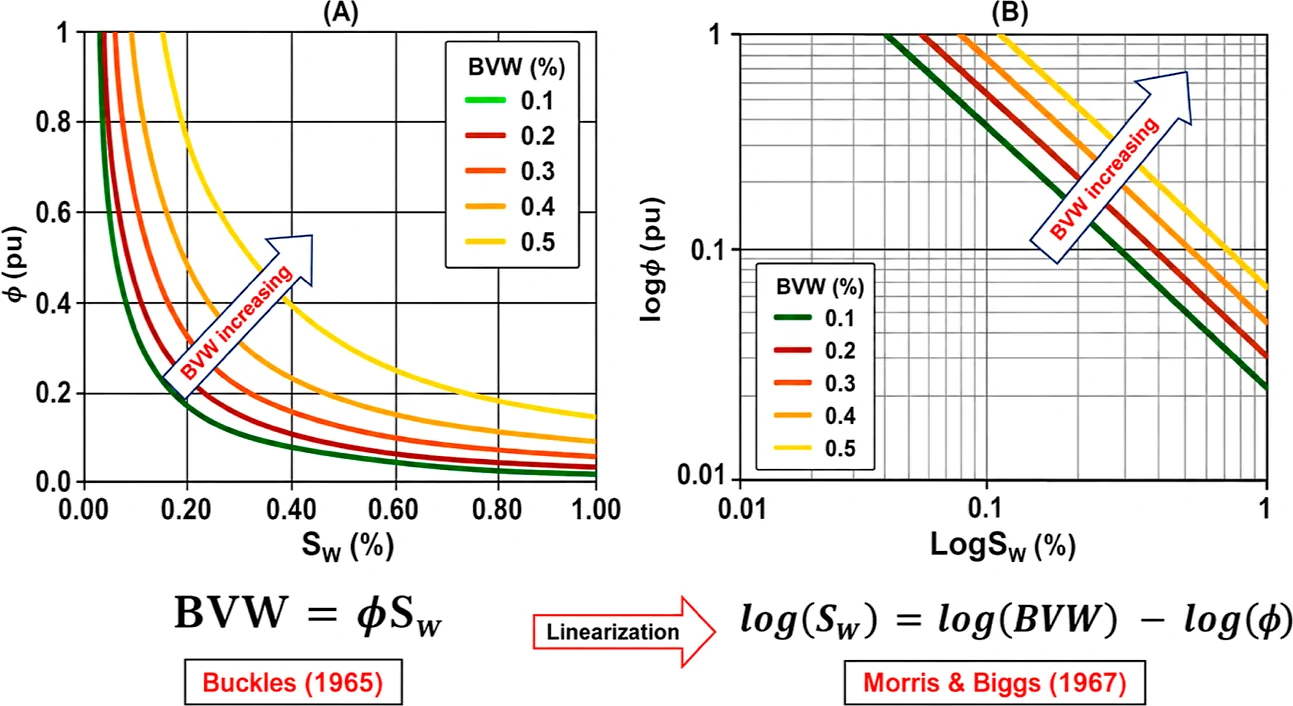
BVW cross-plots showing the relationship between ϕ and S_W_. Adapted from the methods of Buckles^28^ and Morris and Biggs,^29^ and independently compiled and redrawn after Krygowski and Cluff.^30^

The NPHI vs RHOB^32^ cross-plot is a visual tool in petrophysics that helps identify lithology (e.g., sandstone, limestone, dolomite, shale, halite, anhydrite, salt), ϕ, and the presence of gas in subsurface formations, with diagonal lines representing different matrix densities (Figure 7).

**Figure 7:**
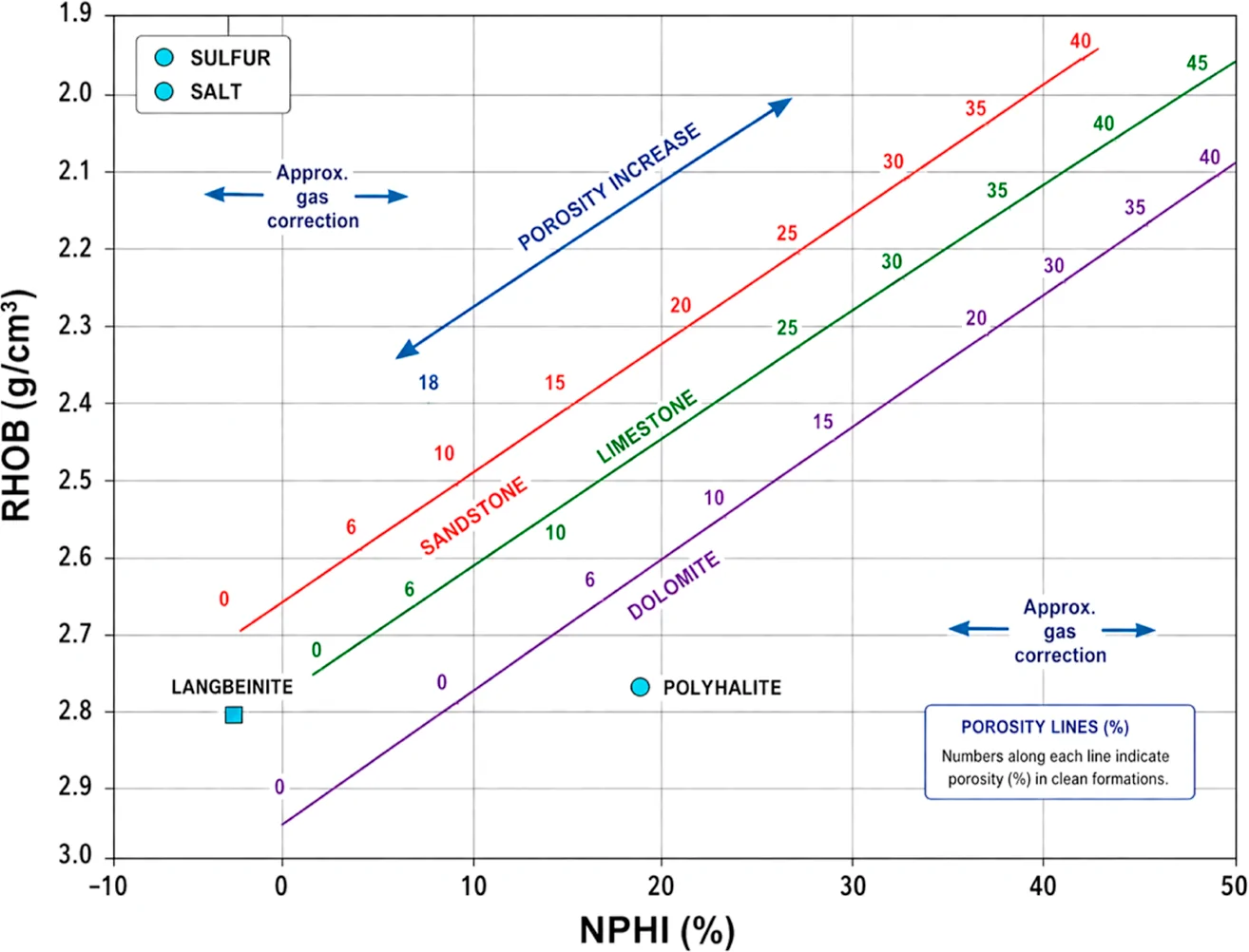
NPHI-RHOB cross-plot used to interpret lithology and porosity. Adapted from the traditional method used in Schlumberger log interpretation and independently compiled and redrawn based on Schlumberger.^32^

The M-N^32^ cross-plot is an analytical tool used in well-log analysis to assess lithology and mineralogy by plotting parameters M and N derived from RHOB, NPHI, and DT log data. This method assists in identifying mineral types such as quartz, dolomite, and shale, as well as in determining mineral proportions and secondary porosity (Figure 8).

**Figure 8:**
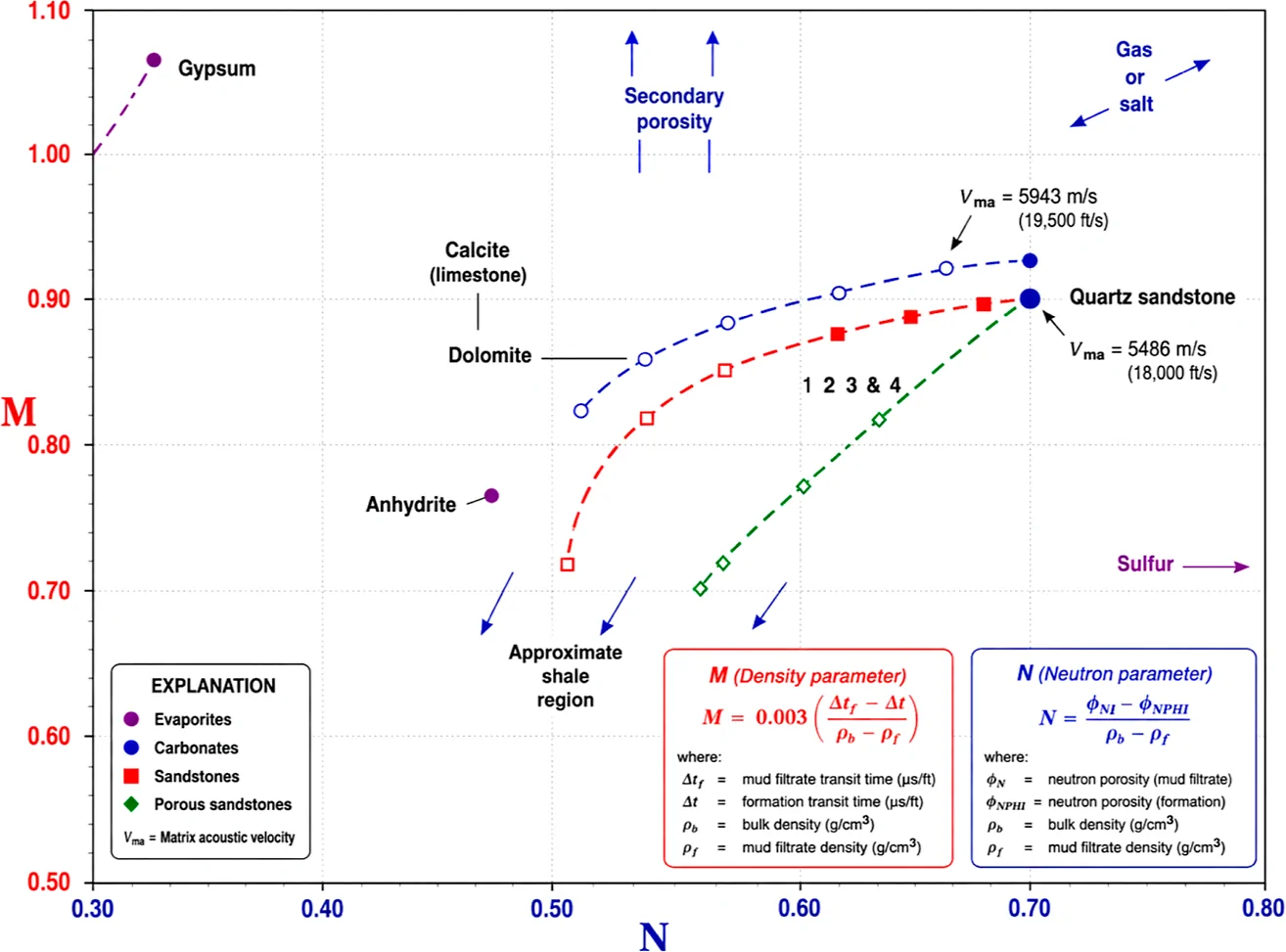
M-N cross-plot for mineral and lithology identification from DT, RHOB, and NPHI log responses. It is adapted from the traditional method based on the M–N interpretation methodology of Schlumberger and was independently compiled and redrawn based on Schlumberger.^32^

The properties derived from various cross-plots are the Hingle^26^ cross-plot, which provides ρ_MA_, DT_MA_, and R_W_; Pickett^27^ provides SW; Buckles^28^ provides BVW; and the NPHI-RHOB^32^ and M-N^32^ cross-plots reveal lithology.

Some parameters were calculated in this work. For example, ϕ was calculated from RHOB and DT logs using eqs 1 and 2, respectively, following the methodology described as

1 $$\phi_{RHOB}=\frac{\rho_{MA}-RHOB}{\rho_{MA}{-\rho }_{FL}}$$

2 $$\phi_{DT}=\frac{DT-{DT}_{MA}}{{DT}_{FL}{-DT}_{MA}}$$

where ρ_MA_ and DT_MA_ are as stated above, ρ_FL_, and DT_FL_ (μs/feet) are the density and the transit time of the fluid saturating the rock.^32^

Equation 3 was used to calculate S_W_

3 $$S_{w}={\left(\frac{a^{*}R_{w}}{{R_{t}}^{*}\phi^{m}}\right)}^{1/n}$$

where m, ϕ, *R*_*W*_, S_W_ are as defined above, and n is the saturation exponent.^31^ The values used for these coefficients are *a* = 1, *m* = 2, *n* = 2, and *R*_*W*_ = 0.01 Ω·m in the case of sandstone reservoirs.^33^

The parameters M and N were calculated using eqs 4 and 5 to prepare the M-N cross-plot, with GR values as the third variable.

4 $$M=\left(0.03\right)\left(\frac{{DT}_{FL}-DT}{RHOB-\rho_{FL}}\right)$$

and

5 $$N=\frac{\phi_{NFL}-NPHI}{RHOB-\rho_{FL}}$$

where DT, DT_FL_, RHOB, ρ_FL_, NPHI are defined above, and ϕ_NFL_ is the neutronic porosity of the liquid, approximately 1.0 dec,^32^

The volume of clay was calculated using the GR log and eq 6, as

6 $$V_{SHALE}=\frac{GR-{GR}_{\mathrm{MIN}}}{{GR}_{\mathrm{MAX}}-{GR}_{\mathrm{MIN}}}$$

where GR, GR_MIN,_ and GR_MAX_ are, respectively, the gamma-ray log, minimum, and maximum values of this log.^34,35^

## Results and Discussion

Interpretations of core samples from Petrobras^23^ and ANP^19^ reports suggest that borehole NA-02 in the Namorado oilfield features alternating layers of sandstone, shale, and limestone. Three lithological groups are identified: (1) shale-dominated intervals with carbonate interbeds, (2) sandstone-rich sections with shale intercalations, and (3) mixed shale-limestone sequences (Figure 9, left). Two promising oil reservoir zones are highlighted:^36^ the first from 3038 to 3057 m depth (black rectangle) and the second from 3092 to 3096 m (red rectangle), both showing low GR log readings around 45° API, indicating clean sandstone with low clay content (Figure 9, track 2). This study focuses on the first reservoir level.

**Figure 9:**
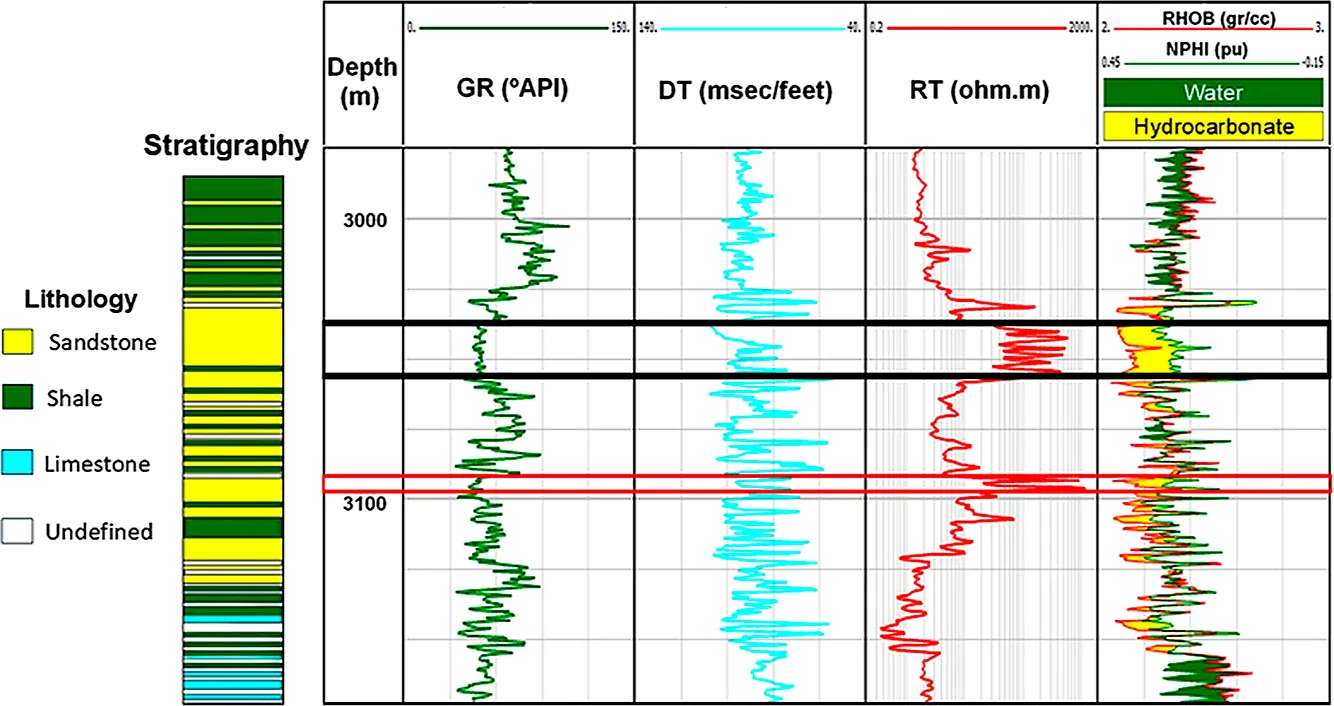
Borehole NA-02 of the Namorado oilfield, featuring the first zone (black), ranging from a depth of 3038 to 3057 m, and the second zone (red), ranging from a depth of 3092 to 3096 m. Tracks: (1) depth (m); (2) GR log­(°API); (3) DT log­(μs/feet); (4) RT log­(Ω·m); (5) RHOB (gr/cm^3^) and NPHI (pu) logs.

Within the reservoir shown in black in Figure 9 (track 3), the DT log increases from 70 to 110 μs/feet, likely indicating higher porosity. The RT log response rises from 2 to 700 Ω·m, suggesting hydrocarbons with high resistivity (track 4). In the interval, the NPHI log increases from 15 to 25 pu, and the RHOB log decreases from 2.3 to 2.1 gr/cm^3^, resulting in a crossover (shaded in yellow) that may indicate hydrocarbons, possibly due to gas. The dark green shading in this track indicates the presence of water.^37^

Porosity (ϕ) calculations in Figure 10 (track 7), derived from RHOB (blue curve) and DT (green curve) logs, indicate an increase from 0.25 to 0.35 pu, surpassing the laboratory porosity range (black dots). S_W_ within the reservoir, calculated using Archie’s equation^31^ with coefficients *a* = 0.81, *m* = 2, and *n* = 2,^36^ is depicted in track 8 of this figure. The results indicate *S*_*W*_ ≤ 0.1 dec, i.e., oil saturation *S*_O_ = 1 – *S*_*W*_ > 0.90 ^37^ dec.

**Figure 10:**
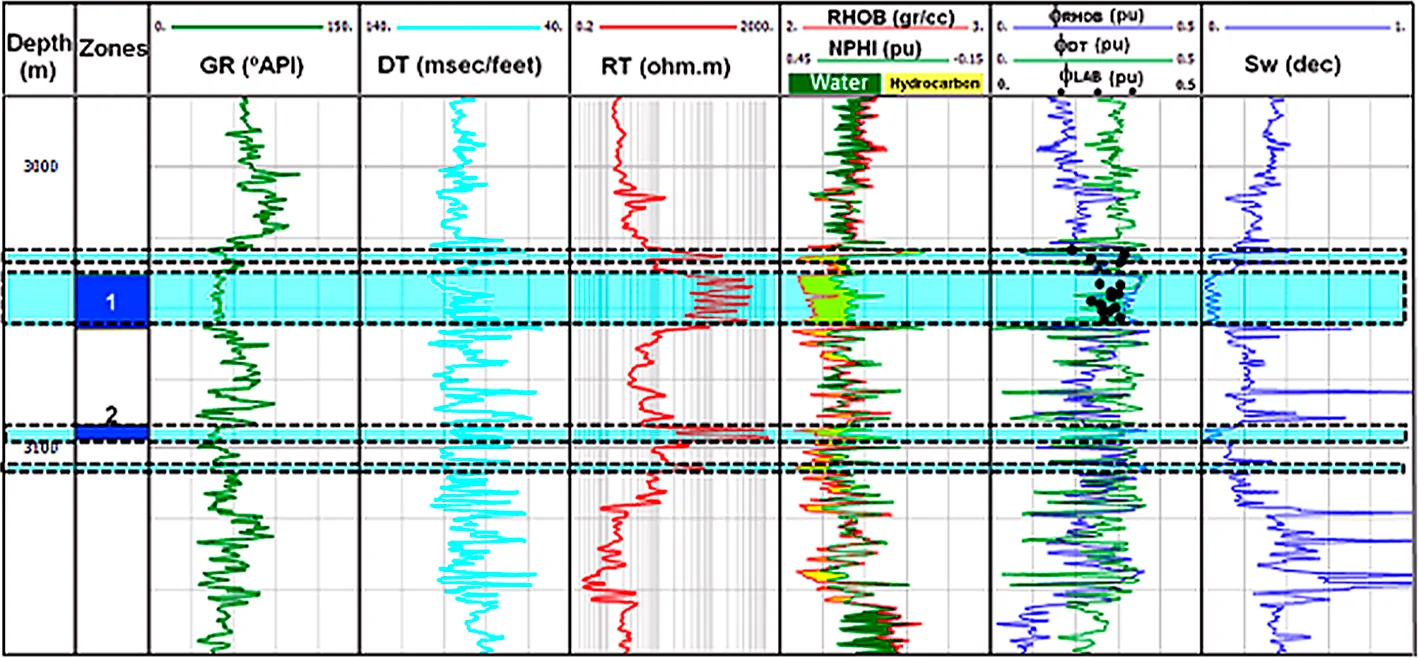
In addition to the NA-02 well logs shown in Figure 9, track 7 shows laboratory porosity (black dots), density porosity (blue curve), and sonic porosity (green curve). Track 8 shows the S_W_ curve (blue) calculated by Archie’s equation.^31^

Both methods show a very strong correlation (*R* = 0.99) with ϕ_LAB_ for porosity, with nearly the same average errors (MAE) of 36.52 and 36.67. Track 6 in Figure 11 offers a detailed view based on this comparison.

**Figure 11:**
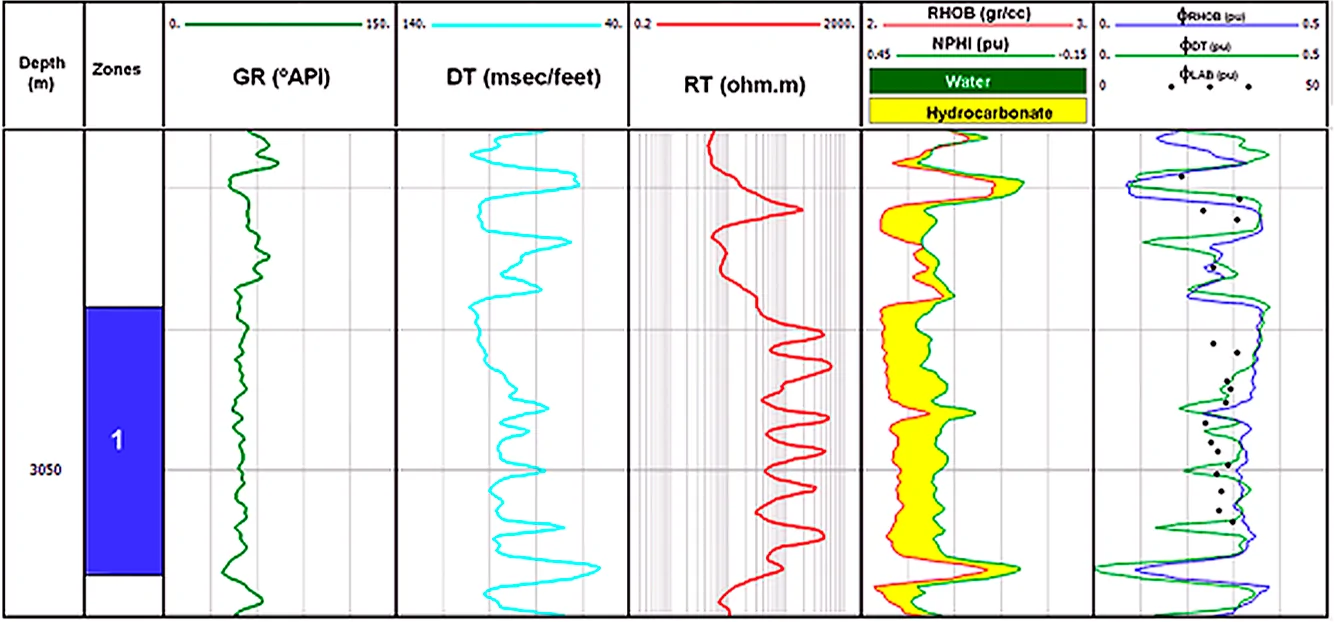
Zoom of the NA-02 well logs in the first zone (dark blue on track 2), alongside the logs shown in Figure 9 (track 7), which display laboratory porosity (black dots), density porosity (blue curve), and sonic porosity (green curve).

The RHOB log measures the total porosity (ϕ_T_) in sedimentary environments because it directly reflects bulk density and is less influenced by compaction and pore shape. The DT log estimates the travel time of sound waves passing through the rock matrix to determine primary porosity (intergranular or intercrystalline), not secondary porosity (vuggy or fractured). On the other hand, laboratory analysis of the core samples evaluates effective porosity (ϕ_LAB_), the interconnected pore space that allows fluid flow, using helium gas expansion or liquid saturation.^38^ The fact that the two porosities are nearly equal indicates that secondary porosity is minimal. Since ϕ_DT_ provides a continuous estimate across the entire well interval, whereas ϕ_LAB_ is derived solely from point samples, ϕ_DT_ was used in subsequent calculations requiring full vertical coverage, supported by its calibration and validation against core data.

After determining that ϕ_DT_ is more appropriate than ϕ_RHOB_, the Hingle^26^ cross-plot was performed using RT, RHOB, and DT logs (Figure 12). The key aspect of this method is to identify the straight line connecting the points of lowest resistivity at a given porosity to the points of highest porosity at a given resistivity. This is the *S*_*W*_ = 1.0 dec line, and its slope determines the formation water resistivity (*R*_*W*_). This same line is used to estimate ρ_MA_ and DT_MA_ at the points where it intersects the horizontal axes, where resistivity becomes infinite, and S_W_ is approximately 0.0 ^36^ dec The results showed *R*_*W*_ = 0.01 Ω·m, estimated from the slope of the line (A). *R*_*w*_ was also calculated using Archie’s equation,^31^ whose values were *a* = 0.81, *m* = 2, and *n* = 2.^36^ It resulted in *R*_*w*_ = 0.0439 Ω·m, close to the value reported by Nacur and Carrasquilla^37^ of *R*_*w*_ = 0.01 Ω·m. The properties of the rock matrix were estimated, with ρ_MA_ = 2.70 gr/cm^3^ (B), and DT_MA_ = 55.00 μs/ft (C), which are near those reported by Stevanato,^39^ who found ρ_MA_ = 2.67 gr/cm^3^ and DT_MA_ = 53.52 μs/ft on average. According to Schön,^33^ the density of sandstone varies from 1.61 to 2.76 gr/cm^3^, depending on porosity and fluid saturation, and the typical matrix (grain) density for quartz-dominated sandstone is 2.65 gr/cm^3^. Certainly, a small ρ_MA_ variation significantly affects ϕ_RHOB_ calculations, typically leading to differences across porosity units.^35^

**Figure 12:**
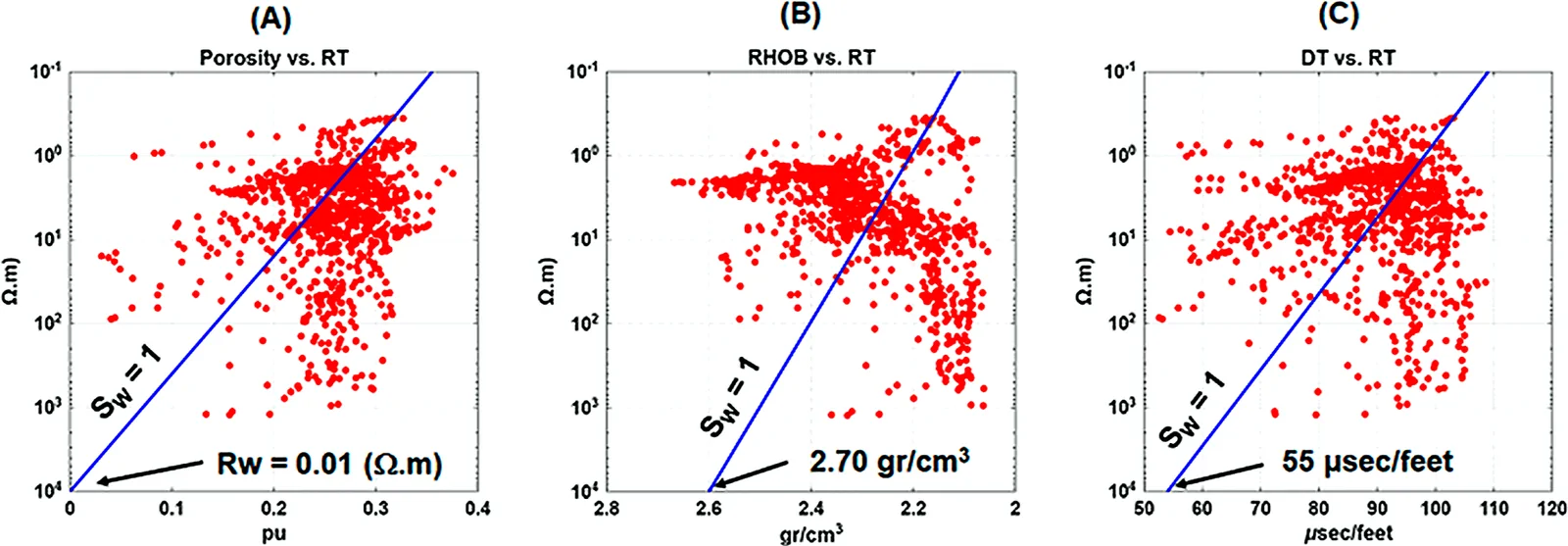
Hingle^26^ cross-plots for well NA-02. (A) Porosity vs RT to estimate S_W_, (B) RHOB vs RT to estimate ρ_MA_, and (C) DT vs RT to calculate DT_MA_.

Using Archie’s equation^31^ to estimate *R*_*W*_, the Pickett^27^ cross-plot was employed to determine Sw, with theoretical lines at 1.0, 0.5, 0.2, and 0.1 dec, based on points from the entire well (Figure 13). The zone with *S*_*w*_ < 0.1 dec was marked with a blue light loop, and the points match the dark blue zones of interest 1 and 2 (Figure 10). The red dashed circle indicates the approximate center of the reservoir, where the average of ϕ is approximately 0.30 pu and the resistivity is about 500 Ω·m.

**Figure 13:**
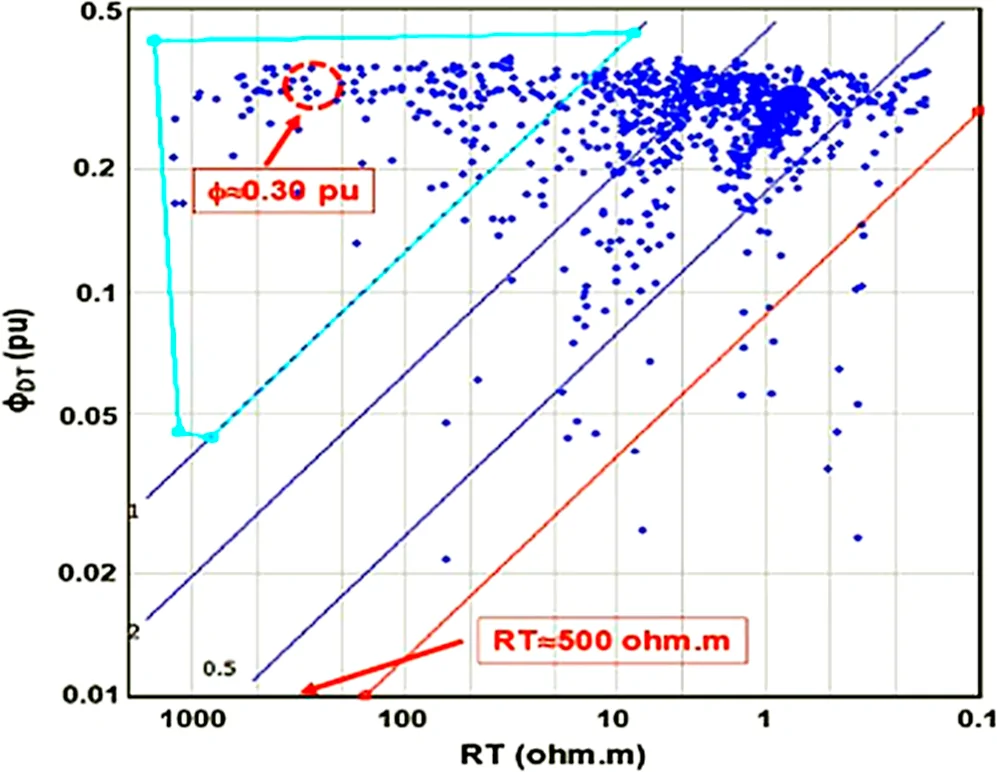
Pickett^27^ cross-plot for well NA-02, estimated using theoretical lines: *S*_*W*_ = 1.0 (red), 0.5, 0.2, and 0.1 dec (all in dark blue). The blue light loop closes the points with *S*_*W*_ ≤ 0.1 dec.

The Buckles^28^ cross-plot was generated using ϕ_DT_, and it was observed that the zone within the red dashed circle in Figure 14 contains little water and more hydrocarbons, with values of *S*_*W*_ ≤ 0.2 dec, 0.01 ≤ BVW ≤0.08 dec, and 0.25 ≤ ϕ_DT_ ≤ 0.30 dec This water is known as irreducible water saturation (S_WIRR_), which, in this case, is approximately 0.01 dec. Capillary forces retain this water, preventing it from moving during oil production. This plot helps distinguish between zones at S_WIRR_ and transition zones where water is mobile.^40^

**Figure 14:**
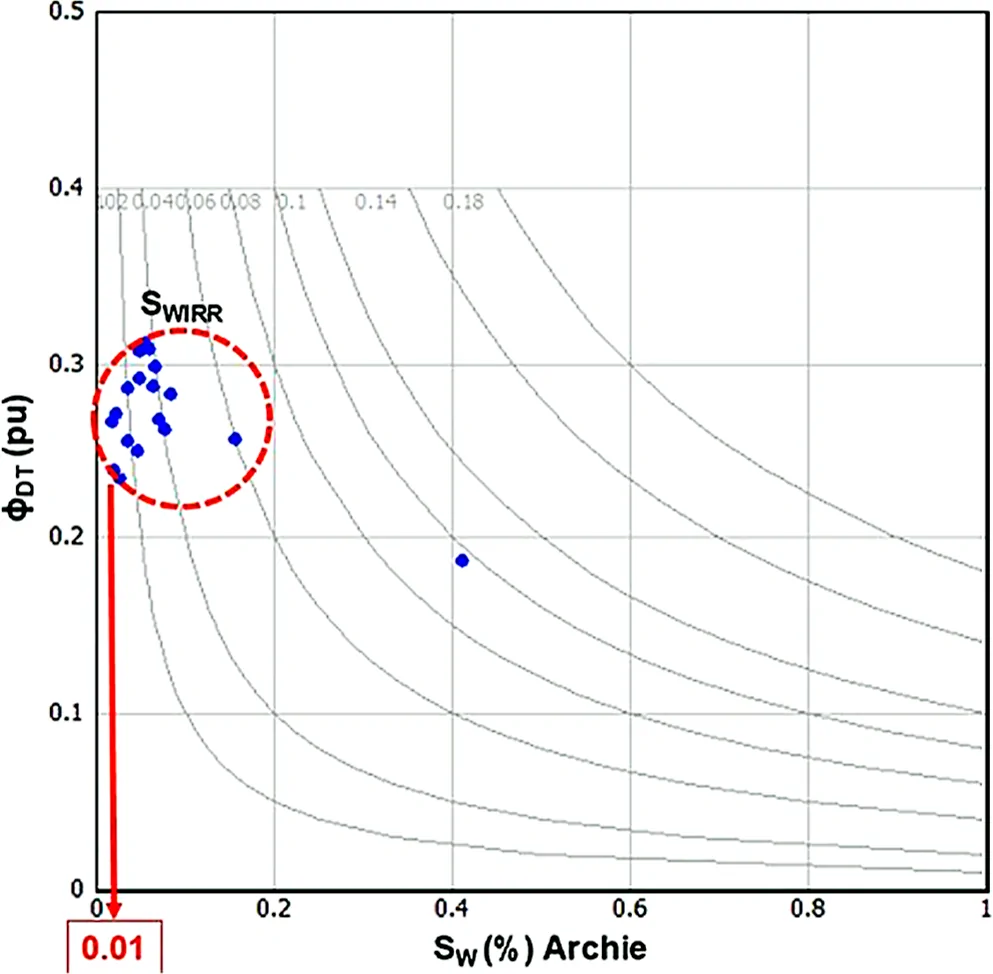
Buckles^28^ cross-plot of well NA-02. The red dashed circle indicates points with *S*_*W*_ ≤ 0.2 dec, 0.01 ≤ BVW ≤0.08 dec, 0.32 ≤ ϕ_DT_ ≤ 0.15 pu, and S_WIRR_≈0.01 dec.

The NPHI-RHOB^32^ cross-plot clearly distinguishes the different rock types and provides important information about the reservoir (Figure 15). In the sandstone (SS) line, a cluster of black points indicates a reservoir porosity of approximately 0.25 dec Considering S_W_ as the third variable in this plot, the S_W_ of this lithology ranges from 0 to 0.1 dec and is associated with increased hydrocarbon concentrations. In the limestone (LS) line, higher S_W_ values (0.1–0.5 dec) with porosity near 0.30 dec, as indicated by the pink and dark blue points, as well as by the presence of light blue points. These light blue points are more abundant between the LS and dolomite (DOL) lines, indicating SW levels of 0.2–0.5 dec and a cluster center (ϕ) at approximately 0.30 ^41^ dec. As noted by Carvalho et al.,^42^ the oilfield has a significant limestone and dolomite content, while the green points in the figure indicate high S_W_ (1.0 dec), though they are limited in number. According to this author, the high porosity of these reservoirs, averaging 0.27 dec, is attributed to the presence of sandstone and to carbonate dissolution.

**Figure 15:**
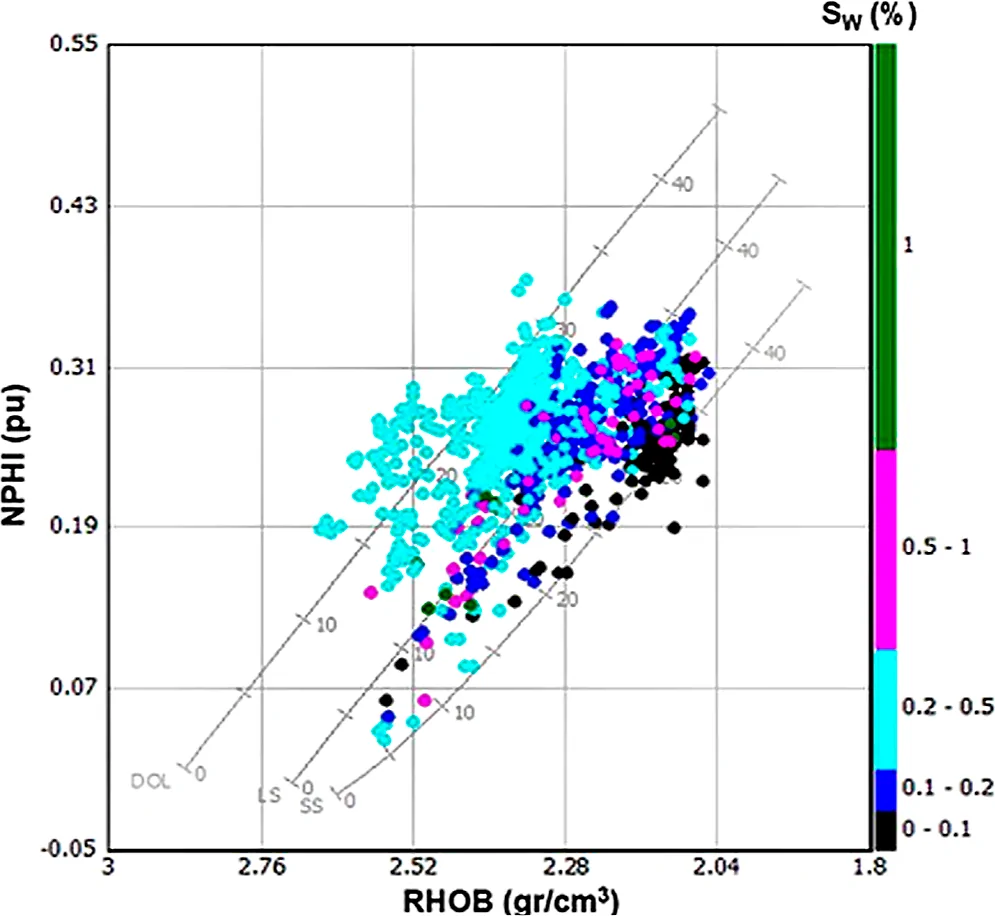
NPHI-RHOB^32^ cross-plot for well NA-02 with S_W_ as a third variable.

The analysis of Figure 16 shows an M-N^32^ cross-plot, with (A) blue circles representing a quartz area with low GR values of 20–50° API, indicating a high-energy formation and strong reservoir potential, corresponding to the sandstone reservoir. The density of this layer (B) ranges from 2.0 to 2.2 gr/cm^3^, which typically has an average density of 2.3–2.7 gr/cm^3^ and a GR of 35°–50° API.^33^ In contrast, the light-green circles represent the calcite group, with GR values of 50°–80° API and densities between 2.2 and 2.4 gr/cm^3^, indicating a medium-energy formation associated with limestone and dolomite, which are poor reservoirs. According to Carrasquilla & Rossi,^41^ they have an average density of 2.7–2.9 gr/cm^3^ and a GR of 10°–40° API. The brown circles indicate the anhydrite-shale zone, which exhibits high GR values (80°–110° API) and density values of 2.4–2.6 gr/cm^3^, comprising formations deposited in a low-energy environment that act as sealing rock and consist of shale and sandstone. According to Carrasquilla & Caetano,^43^ these formations have GR values between 10°–100° API and densities between 1.7–3.3 gr/cm^3^.

**Figure 16:**
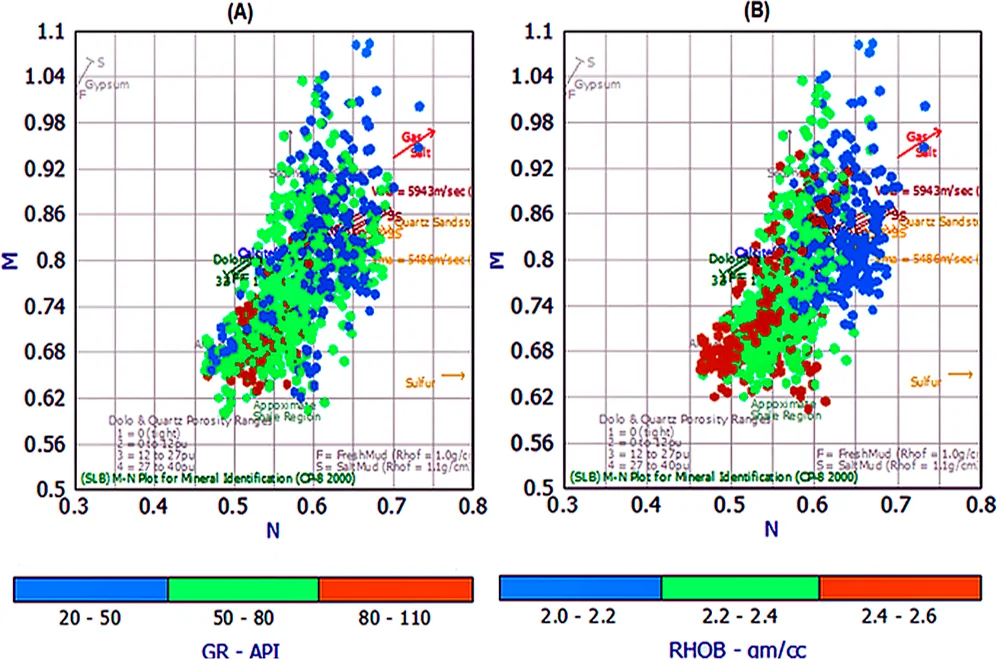
M-N^32^ cross-plot for well NA-02, having as third variables: (A) GR and (B) RHOB logs.

Figure 17 provides a broad classification of the formations discussed above, illustrating the sandstone reservoirs in light green, the shale-sandstone sealing rocks in light blue, and the shale-limestone-poor reservoirs in pink.

**Figure 17:**
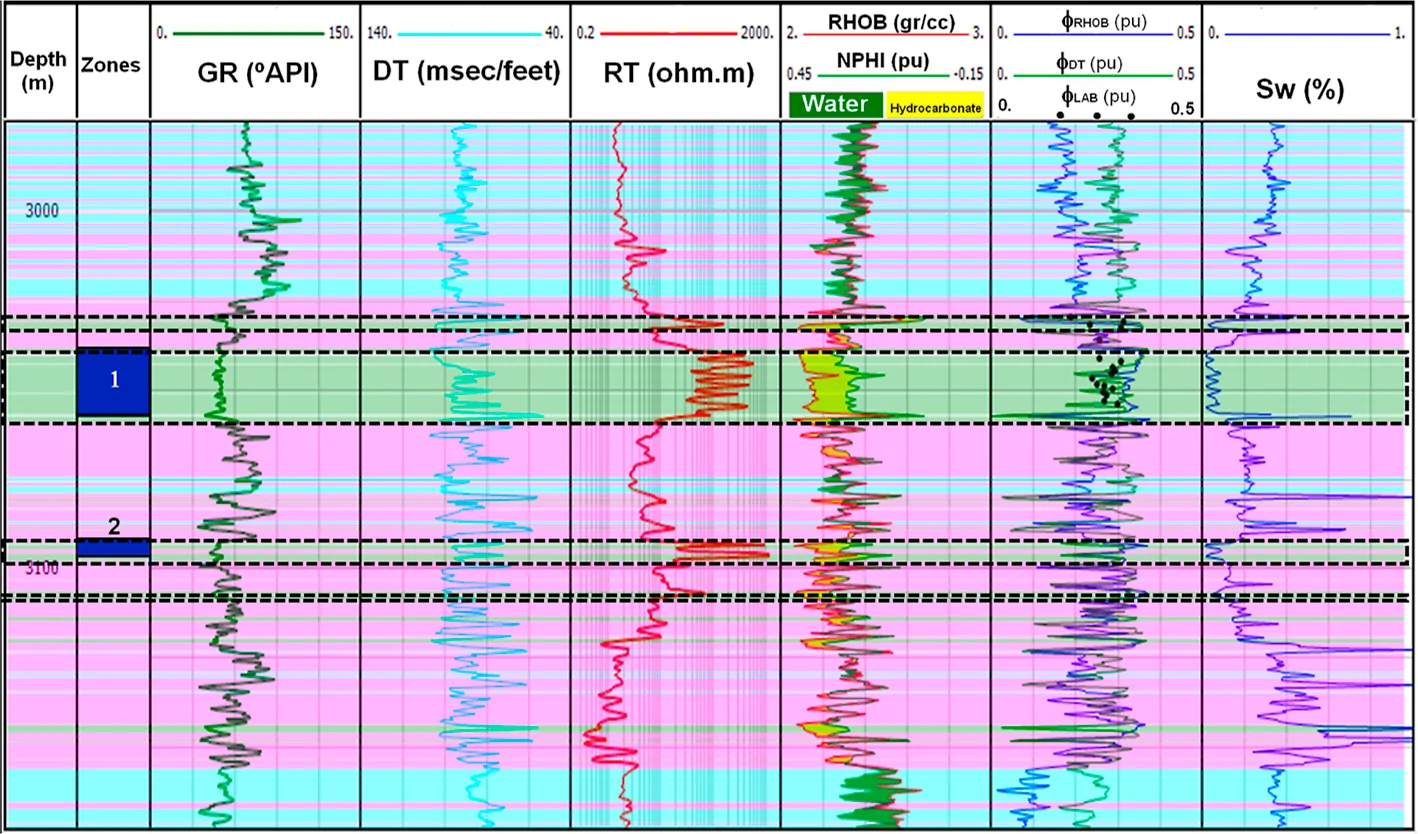
Zones with good reservoir properties (light green), such as sandstone, with shale-sandstone sealing rock characteristics (light blue), and shale-limestone-poor reservoirs.

On the other hand, the stratigraphic column of well EN-17 of the Enchova oilfield in Figure 18 (left) shows layers of grainstones (purple), grainstones/packstones (blue), mudstones (green), and packstones/wackestones (yellow). Grainstones have better reservoir properties, while the combination of packstone, mudstone, and wackestone serves as a seal rock.

**Figure 18:**
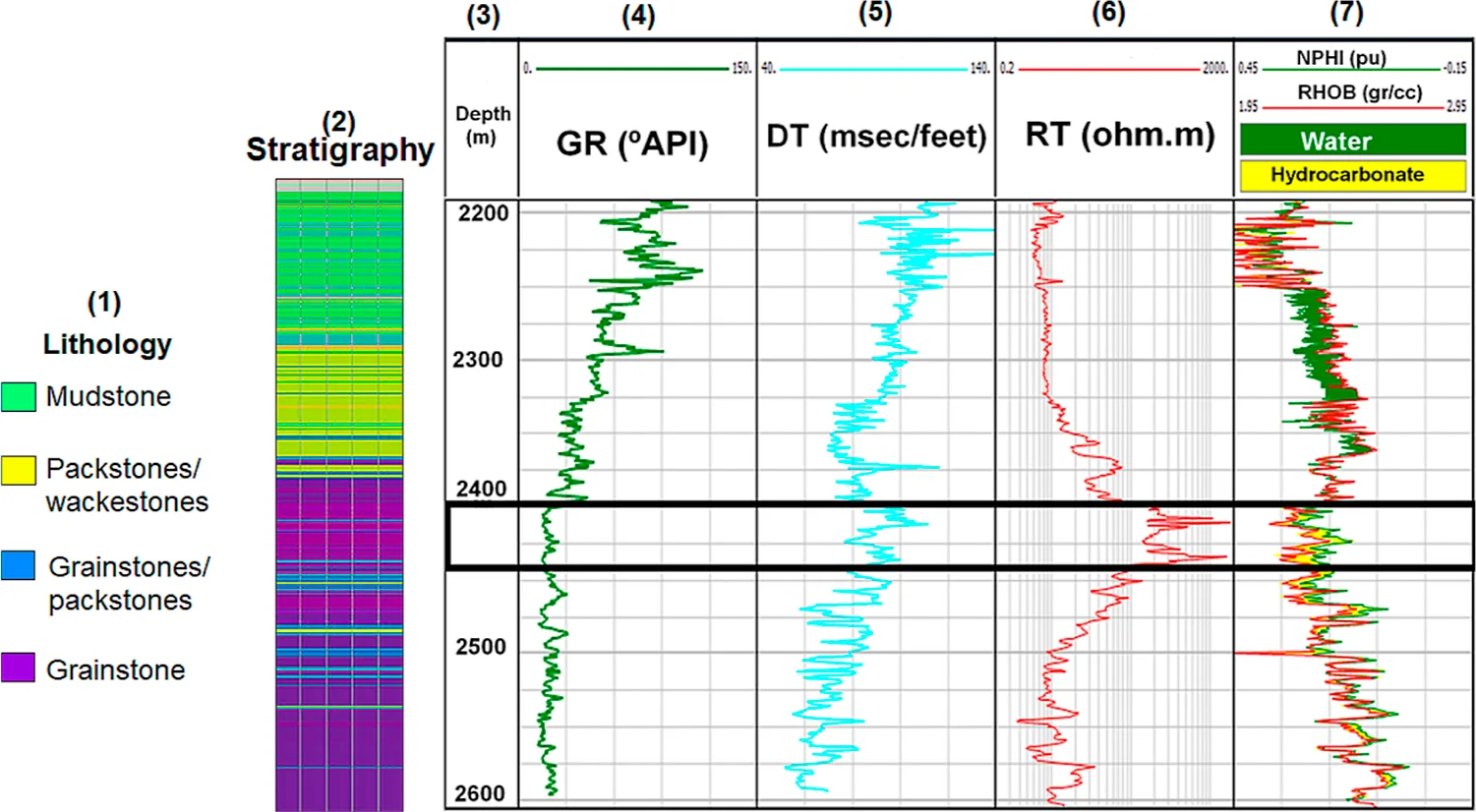
Borehole EN-17 in the Enchova oilfield, featuring an interesting zone ranging from 2400 to 2450 m in black. The tracks are (1) Lithology; (2) Stratigraphy; (3) Depth (m); (4) GR log­(°API); (5) Sonic log­(μs/feet); (6) RT log­(Ω·m); (7) RHOB (gr/cm^3^) and NPHI (dec) logs.

Analysis of these logs shows the GR log decreases from 150° to 20° API around 2325 m (track 1), marking the start of the reservoir interval (track 2). Within this interval, there is a significant drop in the DT log from 90 to 70 μs/feet at 2350 m, probably due to cementation-induced compaction (track 3). The RT logs show a sharp increase from below 10 to over 2000 Ω·m between 2350 and 2460 m, indicating the presence of hydrocarbons (track 4). The RHOB log averages 2.4 gr/cm^3^, typical for limestone, while the NPHI log averages 0.25 to 0.30 pu, decreasing at greater depths (track 5). The combined RHOB and NPHI plot confirms the presence of hydrocarbons (yellow shading), indicating the hydrocarbon-bearing zone between 2350 and 2460 m, with a transition zone extending to 2500 m and an underlying aquifer zone. The dark green shading in this figure indicates the presence of water at depths between 2250 and 2350 m.

When focusing on the interesting zone within the black dashed rectangle in Figure 19, The porosities ϕ_RHOB_ (light blue curve) and ϕ_DT_ (green curve) are plotted alongside the laboratory porosity ϕ_LAB_ (black dots) in track 6. The two generally match ϕ_LAB_, increasing from 0.25 to 0.35 pu, but both exceed ϕ_LAB_, which ranges from 0.25 to 0.30 dec The ϕ_LAB_ aligns more closely with ϕ_DT_ than with ϕ_RHOB_, because although MAE of ϕ_DT_ – ϕ_LAB_ (5.98%) is almost the same as ϕ_RHOB_ · ϕ_LAB_ (5.54), the respective R correlations of 0.55 and 0.44 are more favorable to ϕ_DT_.^43^

**Figure 19:**
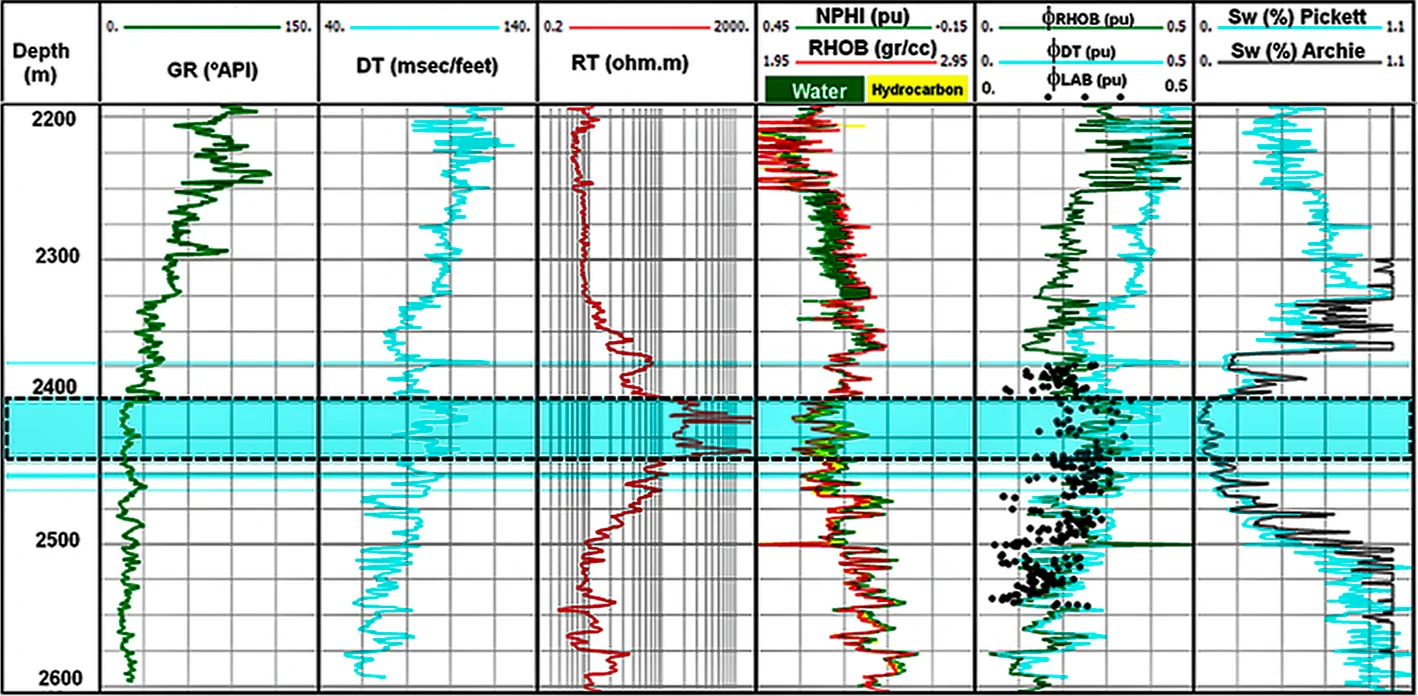
In addition to the EN-17 well logs shown in Figure 18, track 6 shows laboratory porosity (black dots), density porosity (green curve), and sonic porosity (light blue curve).

The black dashed rectangle in Figure 20 (track 6) provides an enlarged view of this comparison. Porosity correlations in the Enchova oilfield appear weaker than those in the Namorado oilfield. The Namorado oilfield data set is a small laboratory data set from the 1970s comprising around 30 reservoir data points. Even if the association is weak or nonexistent, this small sample size may indicate a stronger correlation by chance or due to a few prominent outliers. On the other hand, the Enchova oilfield data set, 10 years older, has about 150 reservoir data points, which may lead to more stable correlations and, therefore, a better approximation of the population value. However, sandstone reservoirs have more stable laboratory measurements than carbonate reservoirs. Sandstones offer a more uniform and predictable porosity structure, making measurements more repeatable and reducing the required sample size. By contrast, carbonates are heterogeneous, making measurements more difficult and erratic and requiring more samples.

**Figure 20:**
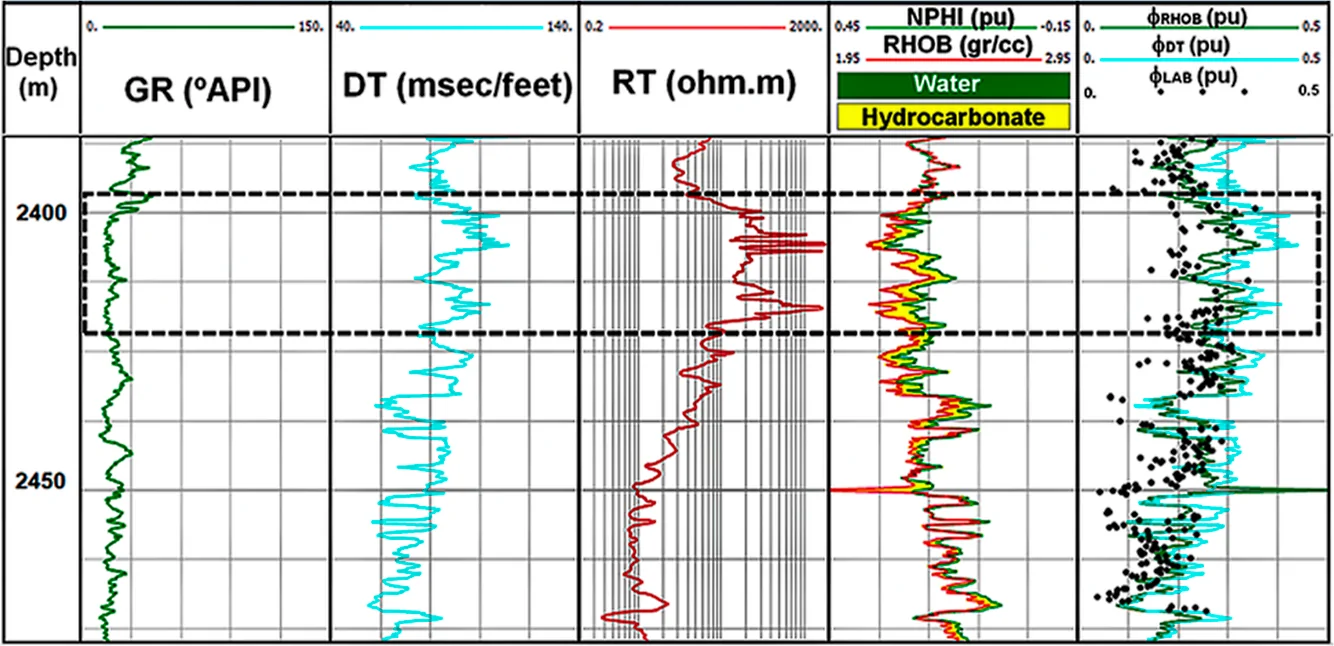
Zoom of the EN-17 well logs shown in Figure 18, along with laboratory porosity (black dots), density porosity (green curve), and sonic porosity (light blue curve) in track 6.

Track 7 in Figure 19 displays the S_W_ curve (blue), calculated using Archie^31^ and Pickett,^25^ with Archie’s equation coefficients *a* = 1, *m* = 2, *n* = 2, and *R*_*W*_ = 0.01, as suggested by Abreu and Carrasquilla.^44^ According to Gomaa et al.,^45^ heterogeneity complicates the estimation of Archie’s parameters in carbonate reservoirs. Laboratory core analysis provides direct and reliable *S*_*W*_ estimates but is time-consuming and costly, and it may lose intervals during coring, so it cannot be applied to all wells in the same oilfield. Instead, default values are often used in reservoir characterization, and continuous well-log data commonly rely on them, which might increase uncertainty in *S*_*W*_ calculations.^46^ Standard Archie’s coefficients, which vary substantially in complex carbonate systems, can lead to uncertainty and severe inaccuracies in water saturation predictions, according to Amin et al.^47^ Still in accordance with Abreu and Carrasquilla,^44^ reservoirs with these standard coefficients are called “Archie reservoirs,” which are clean, homogeneous, water-wet formations (typically sandstones) where S_W_ is calculated using this equation. “Non-Archie reservoirs” (e.g., shaly sands, vuggy carbonates) are heterogeneous or complex and require modified models (e.g., Simandoux, Waxman-Smits, Popon-Leveaux, etc.) due to excessive conductivity from clays or complex pore structures. In the carbonate reservoir case, the same coefficients were used because fine-grained carbonates, or those with homogeneous intergranular porosity, such as grainstones, behave more like clean sandstones and are considered Archie reservoirs.

Thus, S_W_ varies from 1.0 to 0.8 dec between 2200 and 2300 m, decreases from 0.8 to 20 dec between 2300 and 2400 m, and then further drops to less than 0.1 dec between 2400 and 2500 m. It then gradually increases between 2500 and 2600 m, reaching 1.0 dec again (Figure 19). *R*_*W*_ was estimated to use Archie’s equation,^31^ resulting in *R*_*W*_ = 0.028 Ω·m. This aligns with the findings of Vincentelli and Contreras,^48^ who estimated a value of 0.027–0.038 Ω·m in the Albian carbonate reservoir of the Quissamã Formation.

The Hingle^26^ cross-plot of the RT, RHOB, and DT logs showed the following results in Figure 21: (A) *R*_*W*_ = 0.01 Ω·m, (B) ρ_MA_ = 2.70 gr/cm^3^, and (C) DT_MA_ = 54.00 μs/feet. Krygowski & Cluff^30^ state that limestone shows an ρ_MA_ = 2.71 gr/cm^3^ and a DT_MA_ = 47.60–49.0 0 μs/feet, which are approximately the values found. Rw was also calculated using Archie’s equation^31^ and considering this carbonate reservoir as an Archie reservoir, with *a* = 1, *m* = 2, and *n* = 2,^43^ resulting in *R*_*w*_ = 0.0027 Ω. Schmidt et al.^49^ observed that in the Campos Basin, formation water typically has high salinity and resistivities below 1.0 Ω·m in aquifer zones.

**Figure 21:**
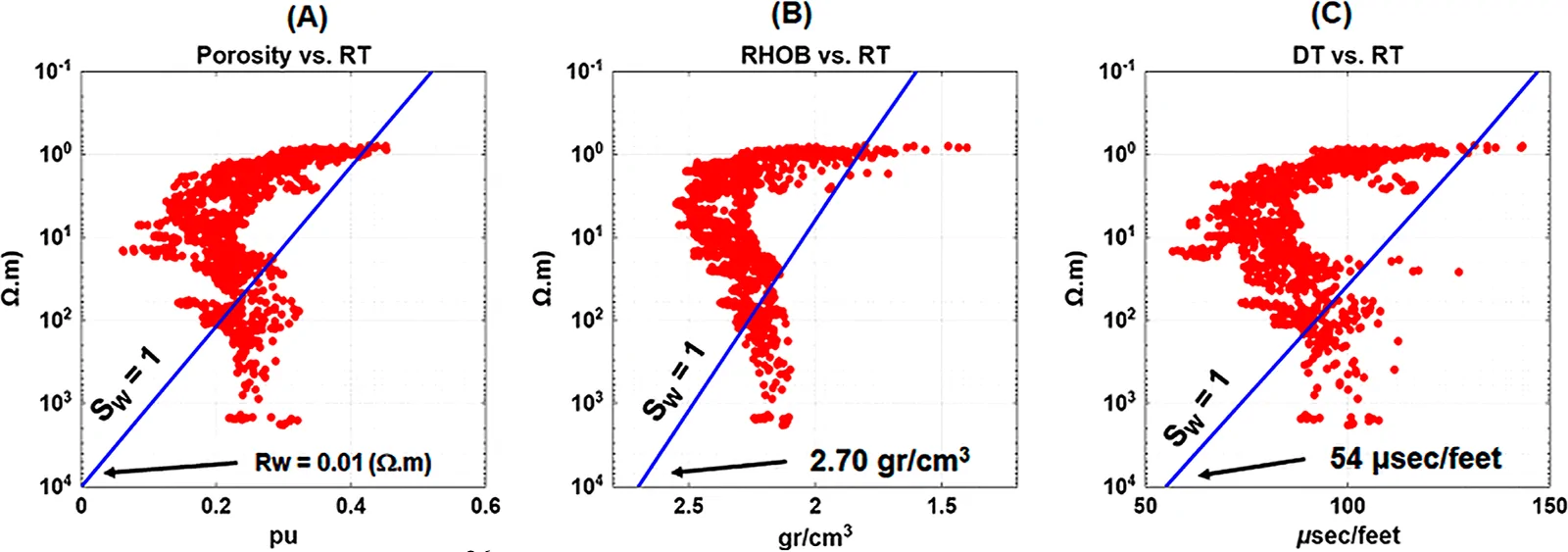
Hingle cross-plots^26^ for well EN-17. (A) Porosity vs RT to estimate *R*_*W*_, (B) RHOB vs RT to estimate ρ_MA_, and (C) DT vs RT to calculate DT_MA_.

Using an *R*_*W*_ of 0.0027 Ω·m, a Pickett^27^ cross-plot was developed displaying S_W_ lines of 1.0 (red) and 0.5, 0.2, and 0.1 (blue) in Figure 22. The area with *S*_*W*_ = 0.1 indicates reduced water content and increased hydrocarbons, as shown by a light blue loop, and aligns with the oil zone. The red dashed circle indicates the approximate center of the reservoir, with a porosity of 0.30–0.35 pu and an RT of approximately 100 Ω·m.

**Figure 22:**
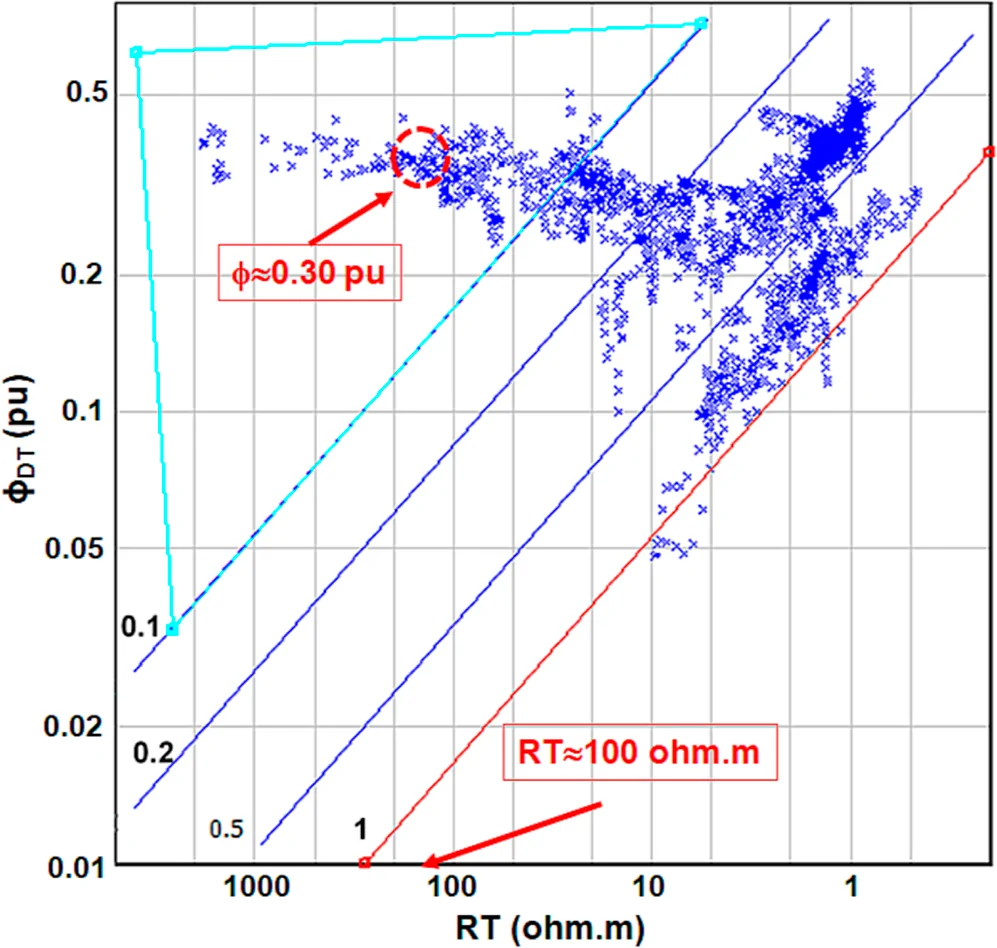
Picket^27^ cross-plot for well EN-17 estimated with theoretical lines *S*_*W*_ = 1.0 (red), and 0.5, 0.2, and 0.1 dec in dark blue. The light blue loop closes the points with *S*_*W*_ ≤ 0.1 dec.

The Buckles^28^ *S*_*W*_ vs ϕ_DT_ cross-plot was created using ϕ_DT_ for well EN-17. The red dashed circle marks points with 0.0 < *S*_*W*_ ≤ 0.2%, 0.00 ≤ BVW ≤0.07, 0.45 ≤ ϕ_DT_ ≤ 0.20%, and S_WIRR_≈0.01 (Figure 23).

**Figure 23:**
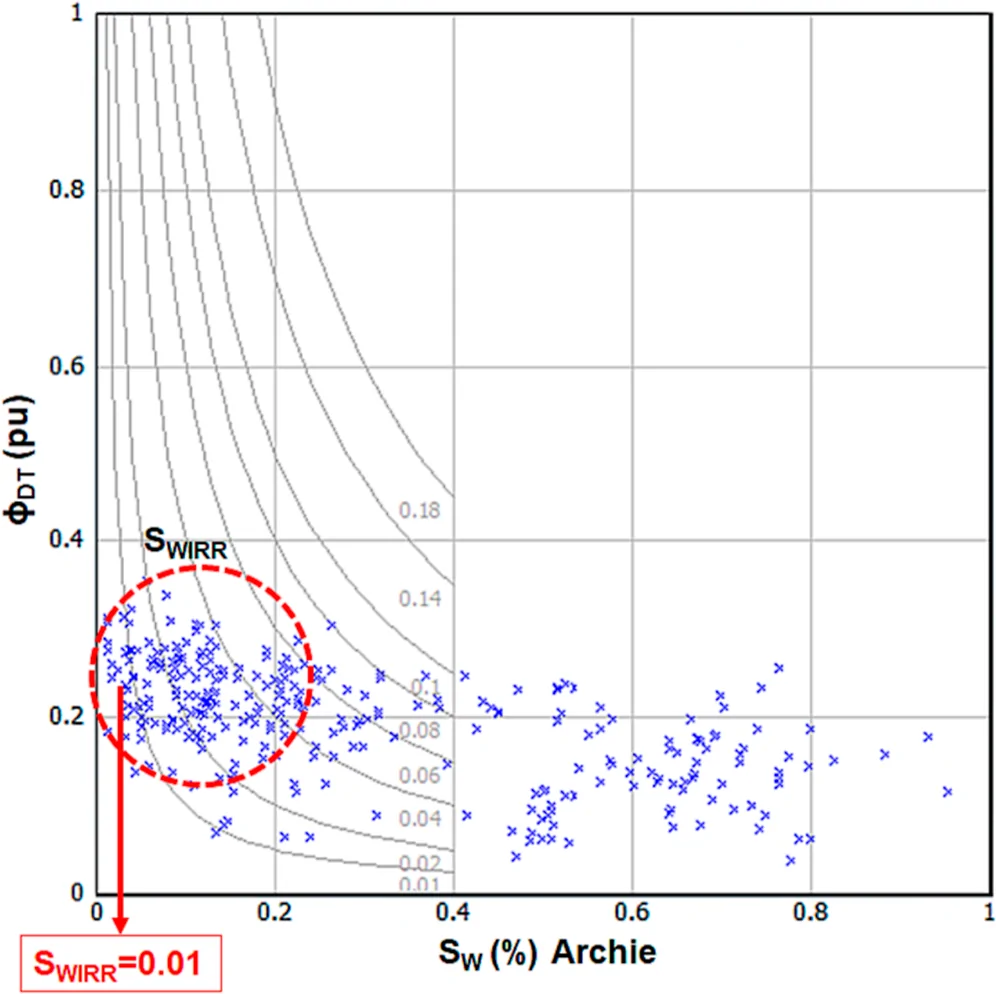
Buckles^28^ *S*_*W*_ vs ϕ_DT_ cross-plot of well EN-17. The red dashed circle indicates points with *S*_*W*_ ≤ 0.2 dec, 0.01 ≤ BVW ≤0.08 dec, 0.35 ≤ ϕ_DT_ ≤ 0.15 pu, and S_WIRR_≈0.01 dec.

Limestone dominates the examined formations, confirmed by the NPHI-RHOB^32^ cross-plot in Figure 24. According to Robaina et al.,^50^ the primary mineral in this reservoir is calcite, with minimal dolomite in low-energy facies. The GR log categorizes energy levels: high (0 to 20 °API), medium (20 to 30 °API), and low (30 to 120 °API). Limestones are prevalent in high- to midenergy zones (blue and light blue), while dolomites are in low-energy areas (pink).

**Figure 24:**
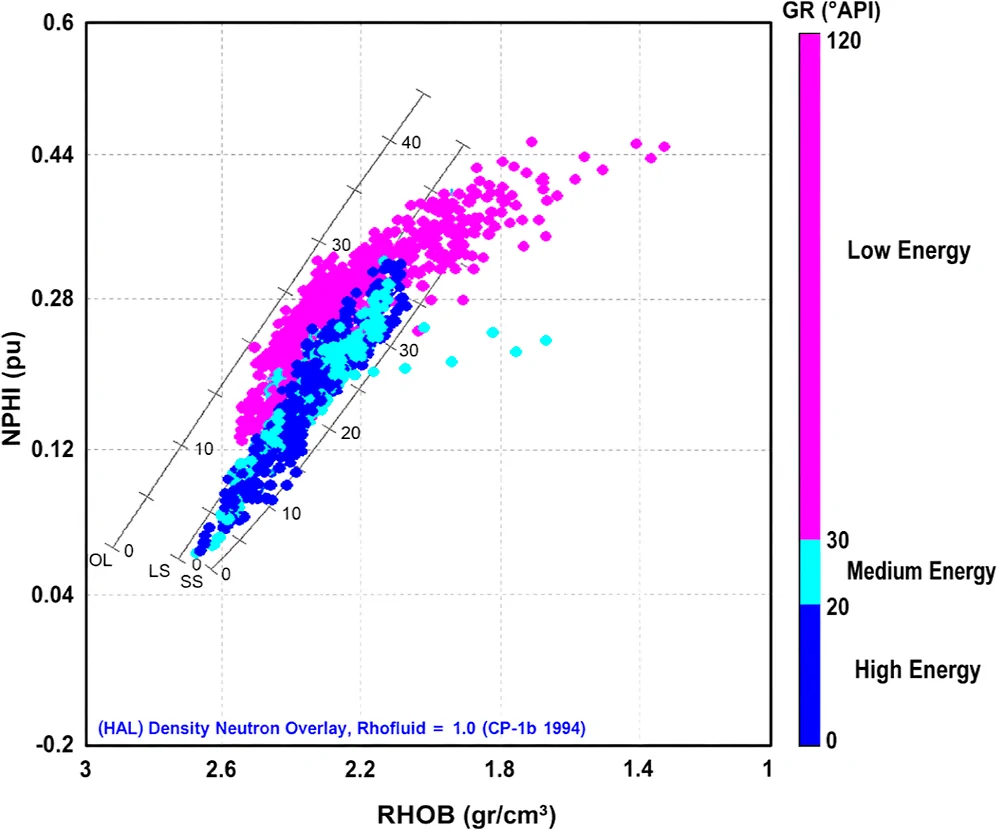
RHOB-NPHI^32^ cross-plot for well EN-17 with GR log as a third variable.

Figure 25 shows an M-N^32^ cross-plot, with (A) blue circles representing a calcite area with low GR values of 10°–50° API, and (B) with RHOB values of 2.2–2.6 gr/cm^3^, corresponding to high- and medium-energy facies that are part of the reservoir zone and consisting of limestone (grainstones/packstones) and dolomite rocks. According to Abreu, C. & Carrasquilla,^44^ when they are porous, they have an average density of 2.2–2.8 gr/cm^3^ and a GR of 10°–40° API. The light-green and brown circles denote the anhydrite-shale zone group, characterized by densities of 2.0–2.6 gr/cm^3^ and GR values of 50°–120° API. These indicate a low-energy formation associated with mudstones, packstones, and wackestones that serve as the sealing rock in the reservoir. According Schön,^33^ they have an average density of 2.0–2.8 gr/cm^3^ and a GR of 10°–150° API. These broad ranges are due to the mixing of the formations.

**Figure 25:**
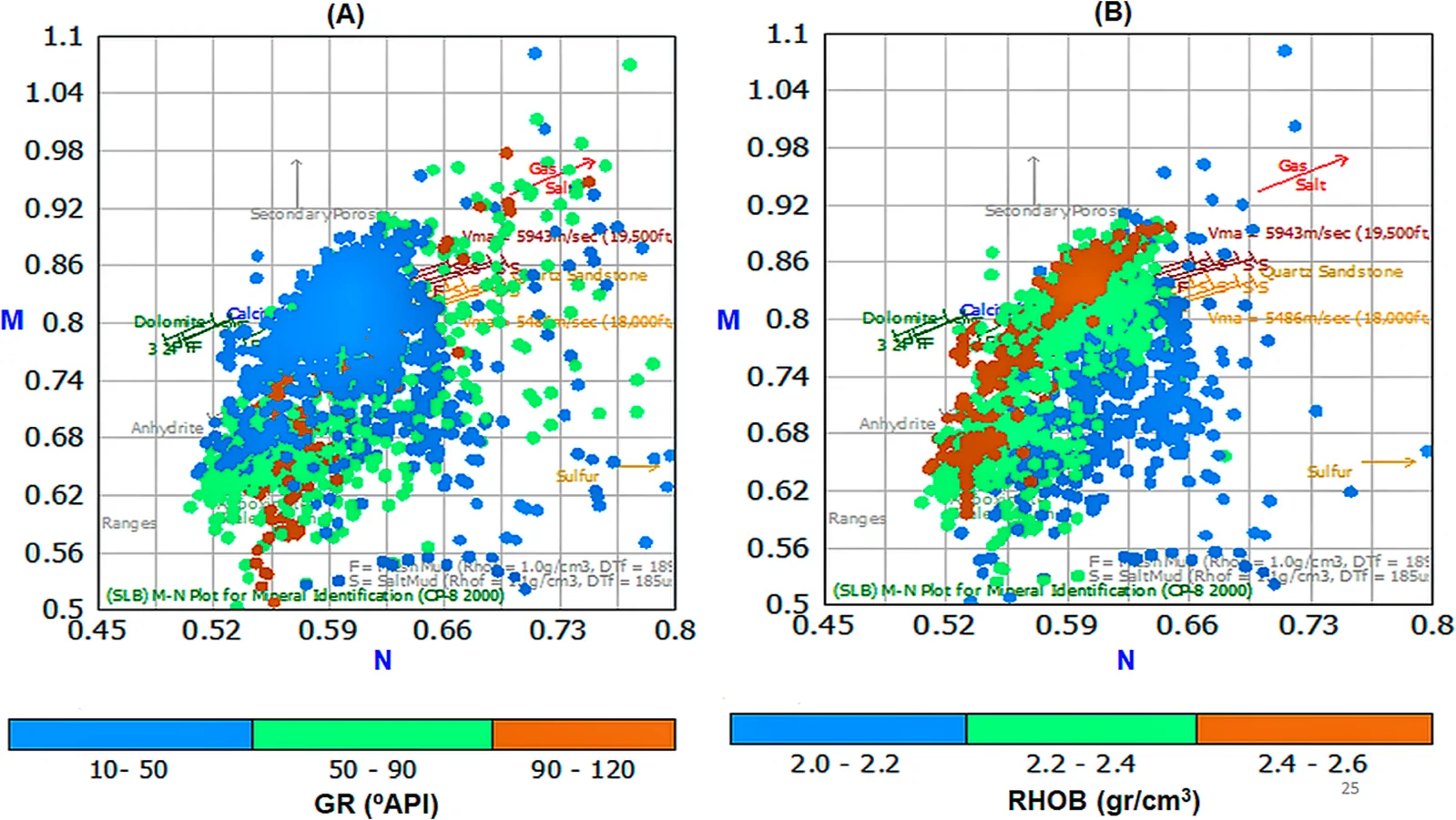
M-N^32^ cross-plot for well EN-17, having as third variable: (A) GR and (B) RHOB logs.

Finally, zones with good reservoir properties (light blue) and those with sealing rock features (pink) are shown in Figure 26. Table 1 summarizes all the results of this study.

**Figure 26:**
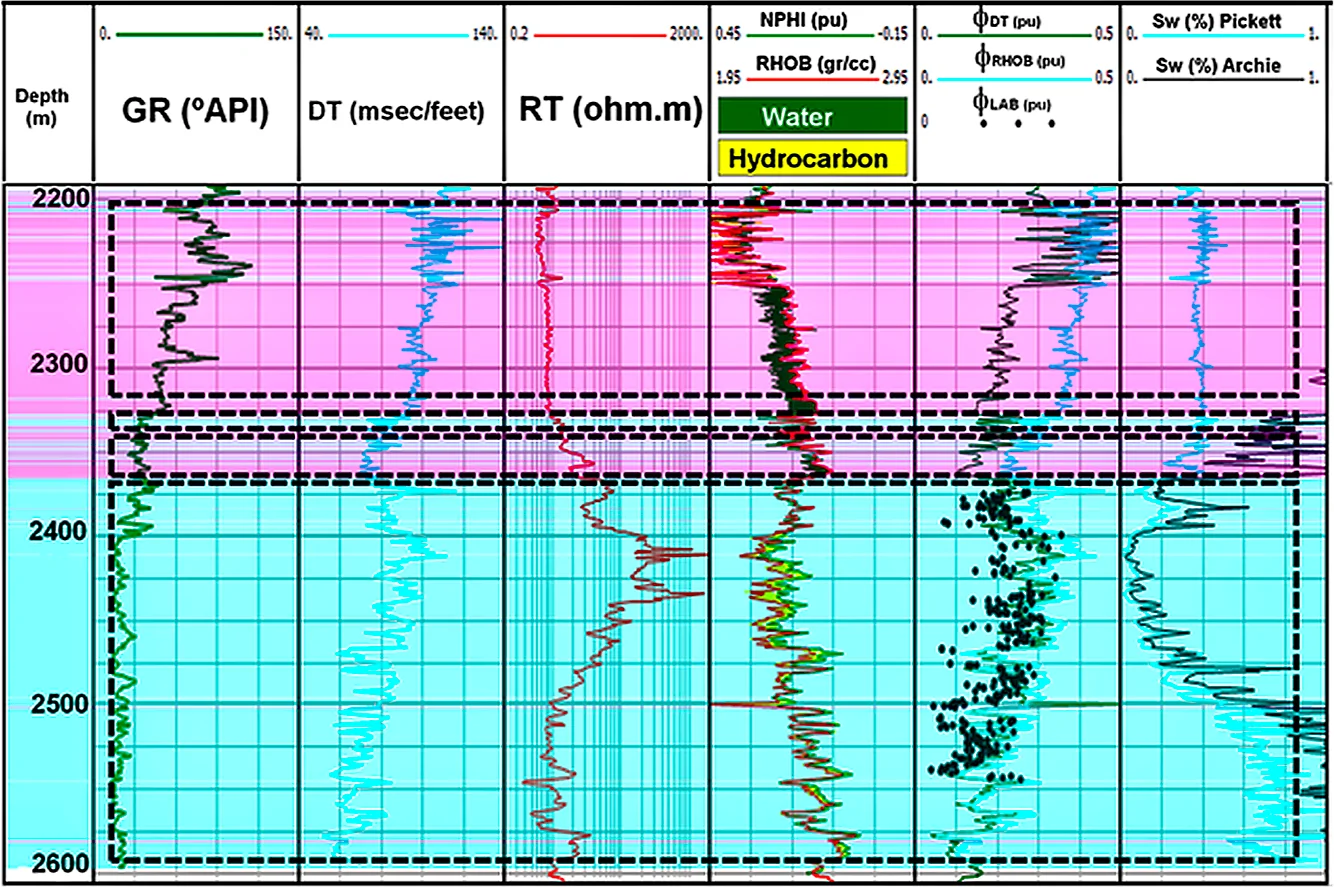
High- and medium-energy zones are highlighted in light blue, and the low-energy zones in pink, in borehole EN-17 of the Enchova oilfield. The logs shown on the different tracks are the same as in Figure 18

**Table 1:** Summary of the Results Obtained for the Reservoir Zones of Namorado and Enchova Oilfields

Property	Namorado	Enchova	Cross-plot
ϕ	0.25–0.30 pu	0.25–0.30 pu	Pickett^27^
ρ_MA_	2.60 gr/cm^3^	2.70 gr/cm^3^	Hingle^26^
Δ*t*_MA_	54.00 μs/feet	55.00 μs/feet	Hingle^26^
*R*_*W*_	0.005 Ω·m	0.0297 Ω·m	Archie^31^
BVW	0.01–0.02 dec	0.0–0.07 dec	Buckles^28^
*S*_*W*_	0.01–0.08 dec	0.0–0.2 dec	Pickett^27^
*S*_WIRR_	0.01 dec	0.01 dec	Buckles^28^
lithology	Sandstone and limestone	Limestone and dolostone	NPHI-RHOB^32^
mineralogy	Quartz, calcite, and shale	Calcite, dolomite, and mudstone	M-N^32^

## Conclusions

This study demonstrates the continued value of geophysical well-log cross-plots for the petrophysical characterization of hydrocarbon reservoirs, using the Namorado and Enchova oilfields in the Campos Basin, southeastern Brazil, as representative examples of siliciclastic and carbonate systems. By integrating core measurements with Hingle, Pickett, Buckles, neutron-density, and M–N cross-plots, the proposed workflow enabled a systematic comparison of reservoirs with contrasting lithological, mineralogical, and fluid-related properties.

The results show that cross-plots provide a rapid and effective means of visualizing relationships among porosity, water saturation, lithology, formation-water resistivity, matrix density, and hydrocarbon potential. The observed differences between the Namorado and Enchova reservoirs confirm that these graphical methods can distinguish reservoir-specific characteristics and support the identification of intervals with favorable petrophysical properties. In addition, the agreement between sonic-derived porosity and available laboratory measurements supports using the sonic log as a continuous porosity indicator across intervals lacking core data. This agreement should, however, be interpreted within the limits imposed by lithology, matrix transit time, compaction, cementation, and reservoir heterogeneity.

Although cross-plot techniques have been used in petrophysics for several decades, the present study highlights their continued relevance when they are applied as part of an integrated and comparative workflow. Their ability to combine conventional well logs, derived petrophysical parameters, and core data makes them valuable for preliminary reservoir screening, interpretation quality control, identification of subtle heterogeneities, and selection of prospective zones. Their graphical nature also facilitates the identification of trends and anomalous data points that may not be readily apparent from analyzing individual logs.

The successful application of the workflow to both siliciclastic and carbonate reservoirs emphasizes the versatility of cross-plots across different geological settings. This flexibility is particularly important in heterogeneous and complex reservoirs, where the response of a single logging tool may be ambiguous. Cross-plots, therefore, provide an efficient framework for integrating complementary measurements and reducing uncertainty in lithological and fluid interpretation.

The results further indicate that conventional cross-plots can complement modern digital and artificial-intelligence-based approaches. They may serve as exploratory tools for selecting relevant variables, as quality-control methods for identifying outliers and inconsistent measurements, and as independent references for validating automated classifications and predictions. Thus, advanced computational methods do not replace cross-plots; instead, they provide a transparent and physically interpretable foundation for data-driven petrophysical workflows.

Overall, this work confirms that integrated cross-plot analysis remains a practical, reliable, and adaptable approach for reservoir characterization. The workflow applied to the Namorado and Enchova fields can be transferred, after appropriate calibration of lithological and fluid parameters, to other sedimentary basins and reservoir types. Its broader application may contribute to more efficient exploration, reservoir evaluation, development planning, and hydrocarbon-resource management.

## Abbreviations

a: tortuosity factor of the Archie equation^21^
ANP: Brazilian National Agency of Petroleum, Natural Gas, and Biofuels
BVW: bulk volume water
DOL: dolomite
DT: acoustic or transit time log
DT_MA_: sonic travel time through the rock matrix
DT_FL_: transit time in formation fluid
EN-17: borehole in the Enchova oilfield
°API: American Petroleum Institute degrees, gamma-ray log measurement
GR: gamma-ray log
gr/cm^3^: measure of density
GR_MAX_: highest value of the GR log
GR_MIN_: minimum value of the GR log
F: formation factor
LS: limestone; m, textural or cementing exponent of the Archie equation^20^
MA: matrix
μs/feet: microseconds per feet travel time log measurement
MR: marine regressive
MT: marine transgressive
M-N: M is the ratio of DT to RHOB logs, and N indicates the ratio of NPHI to RHOB
*n*: saturation coefficient of the Archie equation^20^
NA-02: borehole of the Namorado oilfield
NPHI: neutron porosity log
Ω·m: Ω·m measure of resistivity log
pu: porosity unities
dec: decimal fraction, measure of volume or porosity
ϕ: total porosity
ϕ_LAB_: porosity measured in the laboratory
ϕ_NFL_: neutron porosity of formation fluid
PR: prerift
RHOB: bulk density log
ρ_MA_: rock matrix density
ρ_FL_: fluid density
*R*: rift
RT: true resistivity log
R_W_: water formation resistivity
SC: shallow carbonate
SS: sandstone
*S*_*W*_: water saturation
*S*_*O*_: oil saturation
*S*_WIRR_: irreducible water saturation
*T*: transitional
